# Analysis of putative quadruplex-forming sequences in fungal genomes: novel antifungal targets?

**DOI:** 10.1099/mgen.0.000570

**Published:** 2021-05-06

**Authors:** Emily F. Warner, Natália Bohálová, Václav Brázda, Zoë A. E. Waller, Stefan Bidula

**Affiliations:** ^1^​ School of Clinical Medicine, University of Cambridge, Cambridge, UK; ^2^​ Institute of Biophysics of the Czech Academy of Sciences, Brno, Czechia; ^3^​ Department of Experimental Biology, Faculty of Science, Masaryk University, Brno, Czechia; ^4^​ School of Pharmacy, University College London, London, UK; ^5^​ School of Biological Sciences, University of East Anglia, Norwich, UK; ^†^​ Present address: Ikarovec Limited, Norwich Research Park Innovation Centre, Norwich, UK

**Keywords:** Fungi, *in-silico*, virulence, drug resistance, *Aspergillus fumigatus*, G-quadruplexes, i-motifs

## Abstract

Fungal infections cause >1 million deaths annually and the emergence of antifungal resistance has prompted the exploration for novel antifungal targets. Quadruplexes are four-stranded nucleic acid secondary structures, which can regulate processes such as transcription, translation, replication and recombination. They are also found in genes linked to virulence in microbes, and ligands that bind to quadruplexes can eliminate drug-resistant pathogens. Using a computational approach, we quantified putative quadruplex-forming sequences (PQS) in 1359 genomes across the fungal kingdom and explored their presence in genes related to virulence, drug resistance and biological processes associated with pathogenicity in *Aspergillus fumigatus*. Here we present the largest analysis of PQS in fungi and identify significant heterogeneity of these sequences throughout phyla, genera and species. PQS were genetically conserved in *Aspergillus* spp. and frequently pathogenic species appeared to contain fewer PQS than their lesser/non-pathogenic counterparts. GO-term analysis identified that PQS-containing genes were involved in processes linked with virulence such as zinc ion binding, the biosynthesis of secondary metabolites and regulation of transcription in *A. fumigatus*. Although the genome frequency of PQS was lower in *A. fumigatus*, PQS could be found enriched in genes involved in virulence, and genes upregulated during germination and hypoxia. Moreover, PQS were found in genes involved in drug resistance. Quadruplexes could have important roles within fungal biology and virulence, but their roles require further elucidation.

## Data Summary

The authors confirm that links to access all supporting data, genomes, code and protocols have been provided within the article or are accessible through the supplementary data files. The genomes used were obtained from public repositories (https://doi.org/10.5281/zenodo.3783970; https://mycocosm.jgi.doe.gov/mycocosm/home;ftp://ftp.ncbi.nlm.nih.gov/genomes/).

**Impact Statement**

Fungal infection results in more than 1 million deaths annually and can cause life-threatening illness in the immune compromised; such as those suffering from cancer, tuberculosis, HIV, cystic fibrosis and SARS-CoV-2. Moreover, antifungal drug resistance has emerged as a global threat and we have a critical requirement for new drug targets. Quadruplexes are intriguing four-stranded DNA/RNA structures, which have been implicated in key biological functions and have emerged as potential drug targets to kill drug-resistant micro-organisms. A comprehensive analysis of sequences with the potential to form these structures in fungi has not been conducted to date and their roles within fungi are practically unknown. This article provides the largest analysis of predicted quadruplex-forming sequences in the fungal kingdom and the first in-depth analysis of the location and potential functions of these sequences in a pathogenic fungus. This preliminary screen also unveils quadruplex-forming sequences within virulence and drug-associated genes in the clinically relevant pathogen, *Aspergillus fumigatus*, which could represent novel antifungal targets to explore. We hope this initial study will prompt the generation of interesting ideas within the field and initiate studies which promote the exploration of quadruplexes as potential antifungal targets.

## Introduction

Compared to viruses and bacteria, fungi are underappreciated [1]. They are key contributors to the food and drink, biotechnology and textile industries, whilst also being an important source of novel antimicrobial compounds [2–5]. However, >1.5 million deaths per year are attributed to fungi in humans globally, more than malaria and on par with tuberculosis [6, 7]. Fungi can also cause blindness, serious skin conditions, promote allergic responses and can cause secondary infections in cystic fibrosis, tuberculosis, human immunodeficiency virus (HIV) and SARS-CoV-2 patients [8, 9]. Fungal plant pathogens and oomycetes also destroy around a third of crops annually; enough to feed 600 million people [1]. Whilst fungal infections of amphibians, bats, bees, animals and trees have a huge impact on biodiversity [7]. Thus, fungi are an important asset, but they also pose a devastating global burden, and a deeper understanding of their biology is therefore essential.

The negative effects of fungi are exacerbated by a lack of antifungals and the emergence of multidrug-resistant pathogens with intrinsic resistance such as *Candida auris* and *Lomentospora prolificans* [7, 10]. Current classes of antifungals include azoles (e.g. fluconazole), echinocandins (e.g. caspofungin) and polyenes (e.g. amphotericin B), but resistance to these antifungals is ever more prevalent and there are no vaccines [11]. Indeed, a recent meeting of the World Health Organization (WHO) Expert Group on Identifying Priority Fungal Pathogens highlighted azole-resistant *Aspergillus fumigatus* as one such priority pathogen [12]. Therefore, we have an urgent requirement to identify potential novel antifungal targets.

**Fig. 1.**
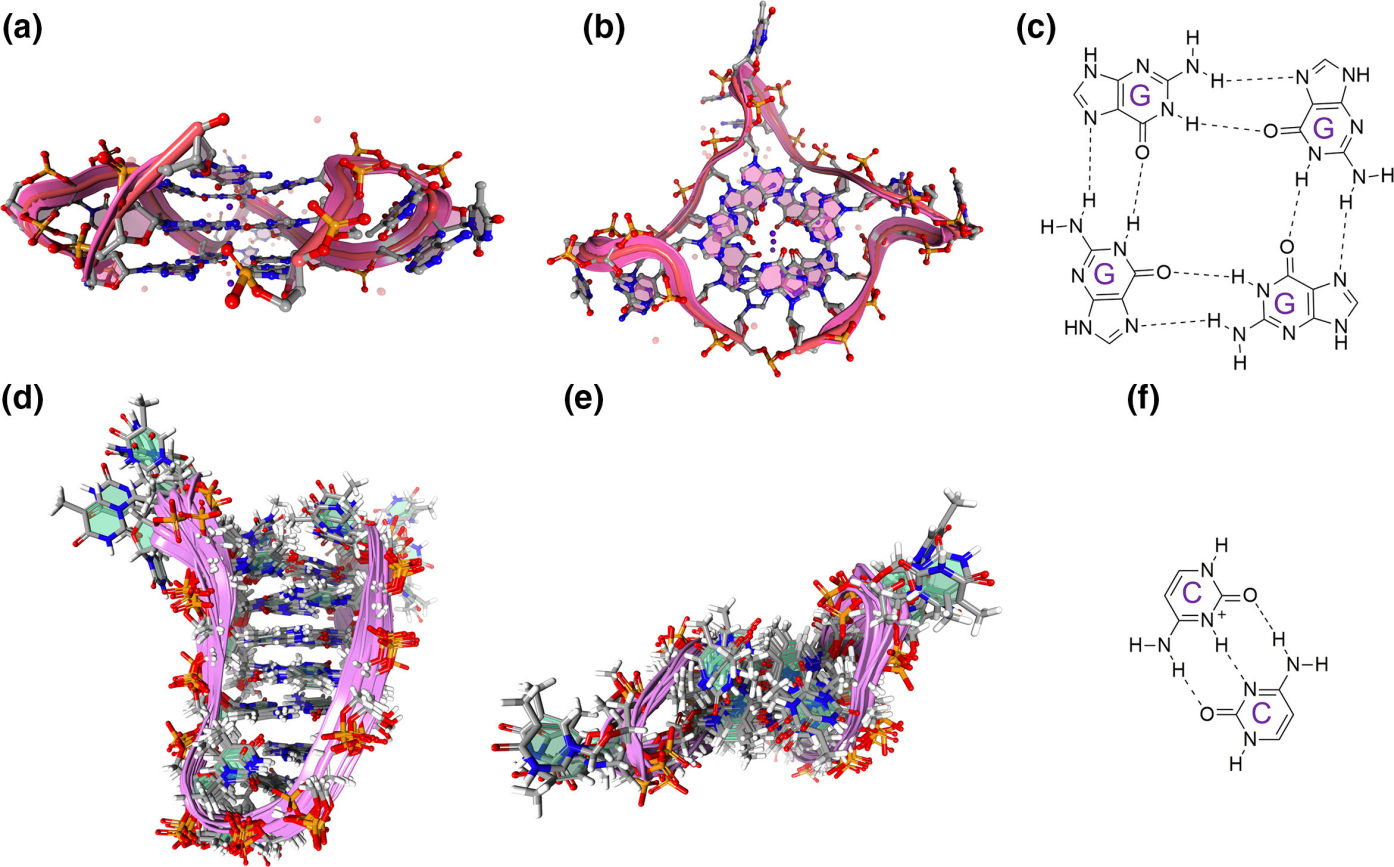
G-quadruplex (G4) and i-motif (iM) structures. Representative examples of G4s and iMs. (a) Side (b) and top-down view of the human telomere DNA quadruplex in K^+^ solution hybrid-1 form (PDB:2HY9). (c) The basic structure of the G-tetrad. (d) Side (e) and top-down view of an intramolecular iM DNA structure with C·C^+^ base pairing (PDB 1A83). (f) C·C^+^ base pairing found in iM structures. Images were generated using the Protein Imager software.

G-quadruplexes (G4s) and i-motifs (iMs) are intriguing four-stranded (quadruplex) secondary structures in nucleic acids that are enriched in regulatory regions, particularly the promoters and telomeric regions of prokaryotic and eukaryotic genomes [13, 14]. G4s can also be found in other segments of DNA and RNA, such as untranslated regions, exons and introns [15]. Here, four guanine bases associate through Hoogsteen hydrogen bonding to form the basic unit of the G4, the G-tetrad [16]. These can then stack on top of each other to form the G4 structure itself (Fig. 1a–e). These stacks of G-tetrads are connected by loops of mixed-sequence nucleotides and can form intramolecular or intermolecular associations [17, 18]. This structure is further stabilized by the presence of monovalent cations, especially potassium [19]. Moreover, the 5ʹ- to 3ʹ-directionality of the strands, glycosidic bonding in the G-tetrads, the cation present, and number of stacked G-tetrads contribute to the wide variation of observed G4 structures and topologies [13]. Conversely, iMs form within cytosine-rich regions of DNA and can typically be found on the complementary strand opposite a G4 [20]. Like G4s, they are four-stranded structures but are composed of two intercalated hairpins, which are stabilized by hemi protonated cytosine-cytosine^+^ (C·C+) base pairs (Fig. 1d–f) [21]. Studies into iMs have been limited compared to G4s as it was thought they were not physiologically relevant based on them being most stable in slightly acidic conditions. However, they have since been shown to form under physiological conditions, including neutral pH and molecular crowding, and have recently been identified within the nuclei of human cells [21–24].

Once deemed structural curiosities, G4s and iMs have been highlighted to participate within host–pathogen interactions, display important roles within gene regulation, and trigger phase separation of RNA in cells [25–27]. However, the regulation of biological functions by quadruplexes is complex and is influenced by their position within DNA/RNA, the surrounding topology, and the environmental factors within the cell. In recent years, there has been increased interest in the therapeutic potential of targeting quadruplexes following the implication of these secondary structures in disease, especially cancer, due to their prevalence in oncogene promoters [26].

There is a growing number of pathogens in which G4s have been shown to contribute to virulence phenotypes, including viruses (human papilloma virus; Epstein–Barr virus, HIV, Adenovirus, Zika virus, Nipah virus and SARS-CoV-2), prokaryotic bacterial pathogens (*Staphylococcus aureus, Streptococcus pneumoniae, Klebsiella pneumoniae, Vibrio cholerae, Salmonella enterica, Enterococcus* spp., *Borrelia* spp.*, Neisseria meningitidis* and *N. gonorrhoeae *), and eukaryotic pathogens (*Trypanosoma brucei*, *Plasmodium falciparum*) [25, 28–34]. Notably, G4 DNA-binding agents have been shown to be active against methicillin-resistant *S. aureus* and vancomycin-resistant *Enterococcus* spp.; potentially providing a novel target to overcome drug resistance [35, 36]. In contrast, iMs have so far only been identified in the long terminal repeat promoter of the HIV-1 pro-viral genome where they modulate the transcription of viral genes [37]. Although, one could assume that this is only the tip of the iceberg, considering the ubiquitous nature of quadruplexes within the genomes of almost every organism.

Practically all organisms possess quadruplexes and due to the advances in the development of drugs that bind and stabilize these structures, the ability to target specific quadruplexes is starting to become a reality [38–40]. A thorough analysis of putative quadruplex-forming sequences (PQS) in fungi has not been conducted to date and could unveil potential drug targets. Here, we identified PQS in publicly available genomes across the fungal kingdom with a focus on the Ascomycota, which contains many known clinically and agriculturally important fungal pathogens. Moreover, we explored and discussed the potential roles of quadruplexes in the human pathogen *A. fumigatus,* and their possible contribution to the virulence of this microorganism.

## Methods

### Selection of genome sequences

The 1107 genome sequences from fungi in the Ascomycota that were analysed in this study were obtained from a publicly available repository (https://doi.org/10.5281/zenodo.3783970). These genomes encompassed the Saccharomycotina (332 genomes from 12 major clades), Pezizomycotina (761 genomes representing 9 of 16 classes) and Taphrinomycotina (14 genomes representing 4 of 5 classes). The completeness of these genomes has been assessed previously [41]. Analysis of the genome completeness based on 1315 full-length Benchmarking Universal Single-Copy Orthologs (BUSCO) genes revealed that 1021/1107 (~92 %) genomes contained >90 % of the 1315 BUSCO genes.

A further 252 published genomes from the Basidiomycota, Mucoromycota, Zoopagomycota, Chytridiomycota, Microsporidia and Cryptomycota were also obtained from the Joint Genome Institute MycoCosm portal (https://mycocosm.jgi.doe.gov/mycocosm/home) and GenBank via FTP access number (ftp://ftp.ncbi.nlm.nih.gov/genomes/). The completeness of these genomes was not assessed, and thus the analysis of these genomes can be found in the Supplementary Material for reference. Information for all the genomes used in this study can be found in Table S1 (available in the online version of this article).

### The principle of the G4Hunter algorithm and process of analysis

The G4Hunter web application was used to identify PQS within fungal genomes [42]. The algorithm used in G4Hunter considers the G-richness and G-skewness of a genome. Each position within a sequence is given a score between −4 and 4, with G’s giving a positive score and C’s giving a negative score. A’s and T’s are neutral and have a score of 0. An increasing G4Hunter score (either positive or negative) correlates with increased propensity to form quadruplexes. A near-zero average score is indicative of a sequence that is most likely to form stable duplexes. The G4Hunter score is the arithmetic mean value of the sequence.

When analysing the genomes, the scored nucleic acid sequence was computed for a sliding window of 30 nucleotides and a threshold above 1.5 for stringent analyses, or a window of 25 nucleotides and thresholds between 1–2 for complete analyses. Regions in which the value of the mean score was above a threshold (either positive or negative) were extracted. The algorithm used specifically recognizes PQS, therefore all sequences obtained from the G4Hunter application can be considered PQS (a mixture of potential G4 and iM sequences).

To identify the location of PQS within annotated genomic features, the file containing the annotations for known genomic features within the genome of the selected fungi were downloaded from the NCBI database. The presence of PQS within a pre-defined genomic feature (e.g. gene, mRNA, mobile element) or within ±100 bp of these genomic features were analysed. Discrepancies in the frequencies of PQS between mRNA and genes are due to how the feature tables downloaded from the NCBI database present their annotations. mRNA length is slightly shorter than the gene length due to the absence of intronic sequences and alternative splicing (e.g. each alternatively spliced mRNA is considered individually by the script).

### Tools used to conduct the analysis

Analysis of the genomes for PQS was conducted using the G4Hunter DNA analyser web application (http://bioinformatics.ibp.cz:8080/#/) [42]. The location of PQS in known genomic features were identified using a publicly available script found at https://pypi.org/project/dna-analyser-ibp/. The protein classes of genes containing PQS were determined via PANTHER Protein Class v.15.0 [43]. Gene ontology (GO)-term analysis of PQS-containing genes was conducted using the FungiFun web tool V2.2.8 (https://elbe.hki-jena.de/fungifun/) and significance was determined via Fisher’s exact test [44]. The PQS-containing genes identified in G4Hunter were verified via another PQS-predictive analysis tool called QGRS Mapper (http://bioinformatics.ramapo.edu/QGRS/analyze.php) [45]. Sequences were analysed using the default settings of max length (30), min G-group (2) and loop size (0–36). The highest scoring sequences with the shortest loop length were selected in each case.

ChemDraw Ultra v12.0 was used to draw structures and The Protein Imager (https://3dproteinimaging.com/protein-imager/) [46] was used to generate 3D images from PDBs. These data were processed, and graphs were generated using GraphPad Prism software v6.01.

### Analysis of transcriptomes

Upregulated genes in germinating conidia and hyphae [47], during hypoxia [48], and during iron limitation or oxidative stress [49] were identified using publicly available transcriptome datasets. These were analysed using the FungiDB transcriptomic resources tool (https://fungidb.org/) [50]. Upregulated genes were identified by comparing a reference sample (control) with a comparison sample (test). In each case, the difference between the minimum expression value of each gene in the control sample and maximum expression value in the test sample was quantified. All genes upregulated >twofold were noted. The identity of genes upregulated >twofold in *A. fumigatus* biofilms [51] were obtained from the Supplementary Material. PQS frequencies in the top 20 most upregulated genes in each condition were identified using G4Hunter and QGRS Mapper using the default search settings. To determine whether the mean frequencies observed for each independent condition were significantly different from the mean frequency observed for the entire genome, outliers were removed using the ROUT method (*Q*=1.0 %) and normality was tested with Shapiro–Wilk, prior to analysis via one-sample *T* test. A *P*-value<0.05 was considered statistically significant.

### Statistical analysis

Statistical analyses were conducted via parametric one-way ANOVA with Tukey’s multiple comparisons, Student’s *T* test, one-sample *T* test or Pearson’s correlation coefficient; or non-parametric Kruskal–Wallis tests with post-hoc Dunn’s test and Bonferroni corrections. Outliers were removed when necessary using the ROUT method (*Q*=1.0 %) and normality was tested using Shapiro–Wilk. All analyses were performed using GraphPad Prism software v6.01. *P*<0.05 were considered statistically significant.

## Results

### There is large heterogeneity in the frequency and number of PQS throughout the fungal kingdom

A thorough analysis of putative quadruplex-forming sequences in fungi has not been conducted to date. Using G4Hunter, the number of PQS were quantified in 1359 genomes across the fungal kingdom. Due to the high variability in genome size and chromosome number between fungal species, to normalize the data, the total number of PQS in addition to the frequency of PQS/kbp and number of PQS relative to the GC content (PQS/GC%) were noted.

**Fig. 2.**
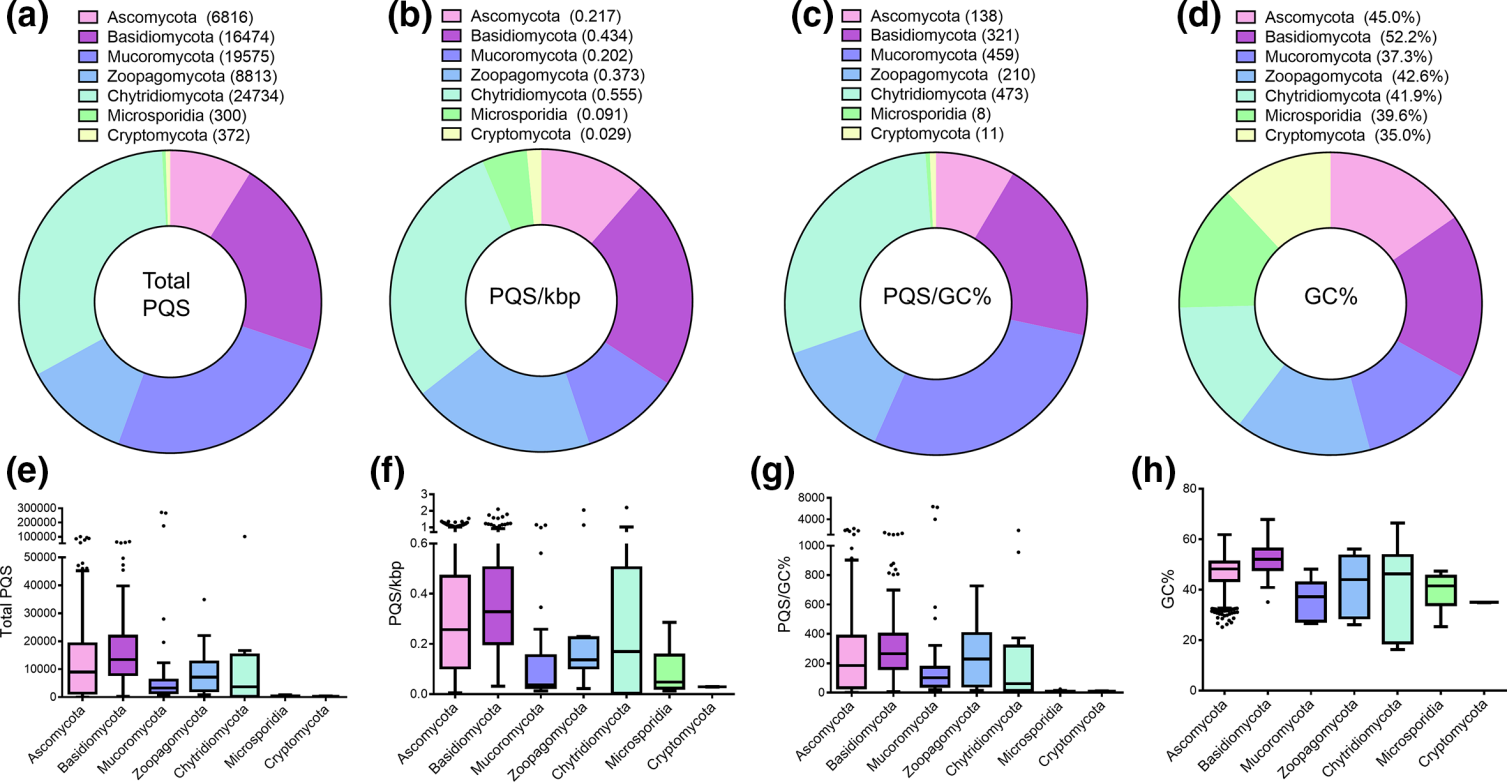
Heterogeneity of PQS across fungal phylum. The total number and frequency of PQS within 1359 fungal genomes were analysed using G4Hunter with a threshold of 1.5 and a window size of 30. The average number of PQS (a, e), PQS/kbp (b, f), PQS/GC% (c, g) and GC content (d, h) in fungi from the Ascomycota (*n*=1107), Basidiomycota (*n*=186), Mucoromycota (*n*=31), Zoopagomycota (*n*=12), Chytrdiomycota (*n*=12), Microsporidia (*n*=9) and Cryptomycota. (*n*=2). (e–h) contain boxplots with Tukey whiskers. The outliers are indicated by dots and the line within the boxplot is representative of the median value.

Across the divisions, the Chytridiomycota had the largest average number of PQS (24 734 PQS) and highest PQS frequencies (0.555 PQS/kbp and 473 PQS/GC%, respectively; Fig. 2). Large numbers of PQS were also found in fungi from the Mucoromycota and Basidiomycota (19 575 and 16 474 PQS, respectively; Fig. 2a, e). The Basidiomycota and Zoopagomycota had the highest PQS frequencies relative to genome size (0.434 and 0.373 PQS/kbp, respectively; Fig. 2b, f). However, the Mucoromycota and Basidiomycota displayed high PQS frequencies relative to GC content (459 and 321 PQS/GC%, respectively; Fig. 2c, g). Fungi within the Basidiomycota had the highest average GC content (52.2 %; Fig. 2d, h). The Microsporidia and Cryptomycota scored lowest for total number of PQS (300 and 372, respectively), PQS/kbp (0.091 and 0.029, respectively) and PQS/GC% (8 and 11, respectively; Fig. 2). Moreover, they also had low GC content (39.6 and 35.0 %, respectively).

### Fungi from the Pezizomycotina have the highest average PQS frequencies in the Ascomycota

**Fig. 3.**
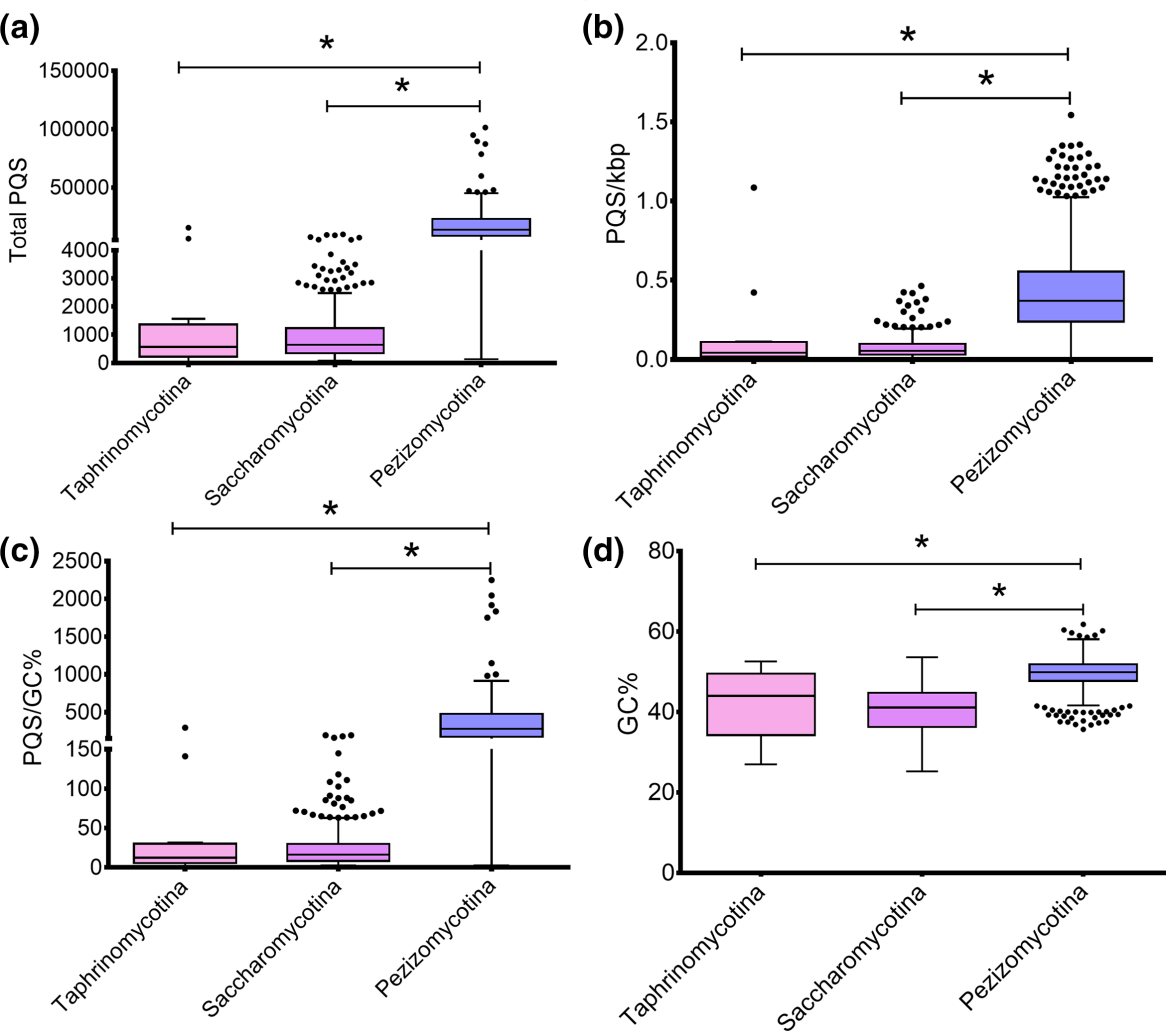
PQS within the sub-divisions of the phylum Ascomycota. The total number and frequency of PQS within fungal sub-divisions of the phylum Ascomycota were quantified using G4Hunter with a threshold of 1.5 and window size of 30. The total number of PQS (a), PQS/kbp (b), PQS/GC% (c) and GC content (d) in fungi from the Taphrinomycotina (*n*=14), Saccharomycotina (*n*=332) and Pezizomycotina (*n*=761). (a–d) contain boxplots with Tukey whiskers. The outliers are indicated by dots and the line within the boxplot is representative of the median value. Significance was determined by one-way ANOVA with multiple comparisons.

More than 1000 of the genomes investigated were from the Ascomycota, and because this phylum contains many of the most prevalent fungal pathogens and ~92 % of the ascomycete genomes contained >90 % of the 1315 BUSCO genes, this phylum was investigated in greater detail. Further analysis identified that there was significant heterogeneity in the total numbers and frequency of PQS between the sub-divisions Taphrinomycotina, Saccharomycotina and Pezizomycotina (Fig. 3). Fungi from the Saccharomycotina had the fewest total PQS on average (1146 PQS), whereas fungi from the Pezizomycotina contained significantly more (17021 PQS). Moreover, this pattern was observed for PQS frequency (0.078/kbp vs. 0.436/kbp), GC content (40.6 vs. 49.6 %), and PQS frequency relative to the GC content (27/GC % vs. 344/GC %) for the Saccharomycotina and Pezizomycotina, respectively (Fig. 3). A complete breakdown of the G4Hunter results for all fungi can be found in Table S2.

**Fig. 4.**
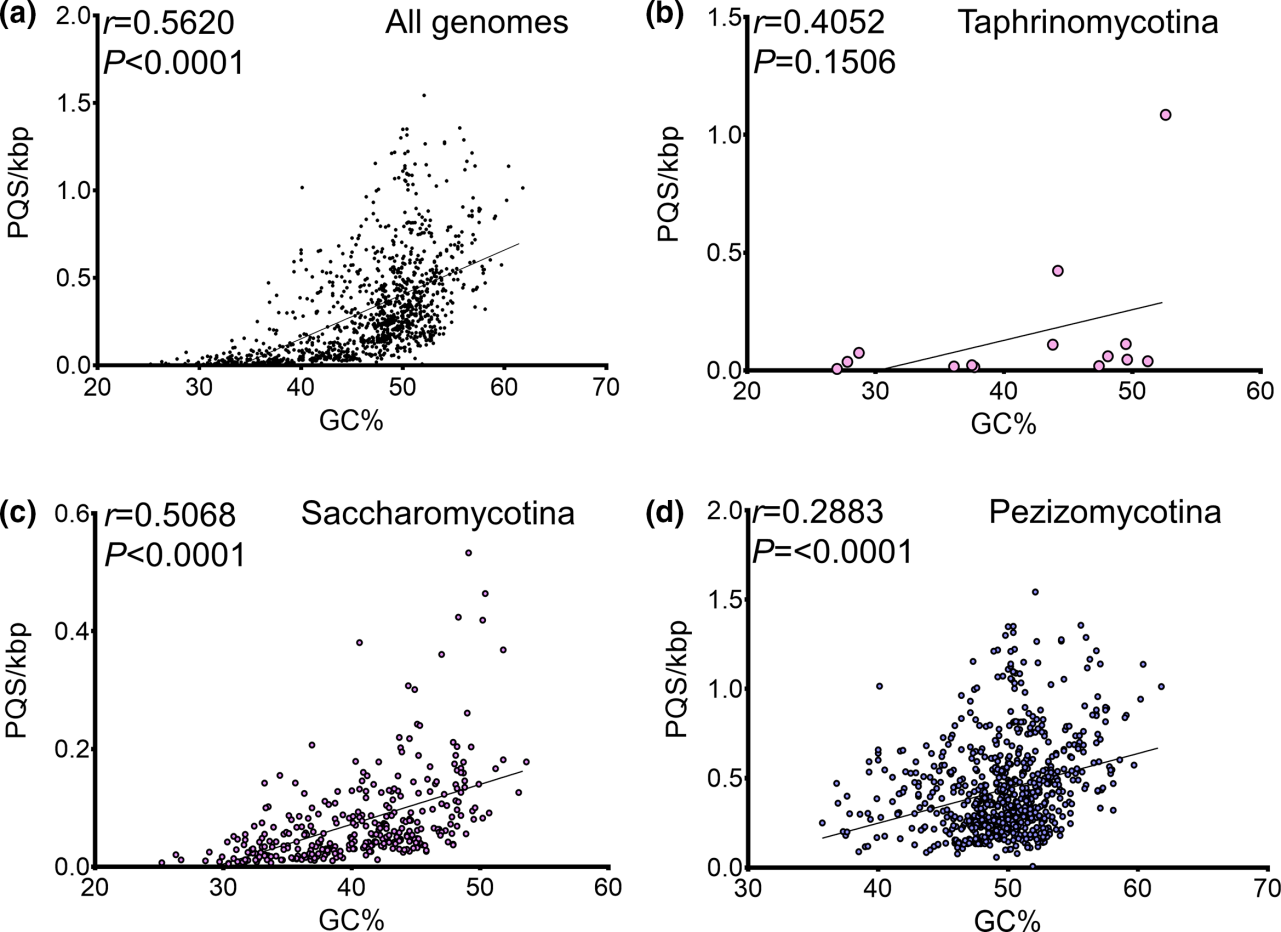
Higher genome GC-content is positively correlated with the frequency of PQS. The frequency of PQS relative to the GC content of fungi was plotted for (a) all fungal genomes in the Ascomycota, (b) Taphrinomycotina, (c) Saccharomycotina, (d) and the Pezizomycotina. The Pearson correlation coefficient was used to determine the association between PQS and GC content. *P*<0.05 was considered statistically significant.

Considering G4s and iMs form in guanine or cytosine-rich regions, respectively, one would expect fungi with a higher genome GC content to have a higher PQS frequency by chance. To investigate this further, the frequency of PQS/kbp relative to the GC content in all fungi were plotted. As expected, there was a positive correlation between GC content and PQS frequency amongst all the ascomycete genomes analysed (*r*=0.5620; *P*<0.0001; Fig. 4a). Moreover, this positive correlation was observed for the Saccharomycotina and Pezizomycotina (*r*=0.5068 and 0.2883, respectively; both *P*<0.0001; Fig. 4b, c). However, the genomes with the highest GC contents did not contain the greatest frequency of PQS, so this association cannot be taken at face value. This correlation was not seen in the Taphrinomycotina, but this is likely due to the much lower number of genomes used (*r*=0.4052, *P*>0.05; Fig. 4d). All the analyses above were also conducted in genomes from the Basidiomycota, Mucuromycota, Zoopagomycota, Chytridiomycota, Microsporidia and Cryptomycota. However, due to their unknown genome completeness, these analyses can be found in Figs S1 and S2.

### PQS are evolutionarily conserved in *Aspergillus* spp.

Evolutionary conservation of genetic motifs within the genome are a hallmark of their fundamental importance to how that organism functions. Therefore, we endeavoured to explore whether there was evolutionary conservation of PQS within fungal genomes. We chose to explore this relationship in *Aspergillus* spp.*,* due to the robustness and accuracy of the phylogenetic tree available [52].

**Fig. 5.**
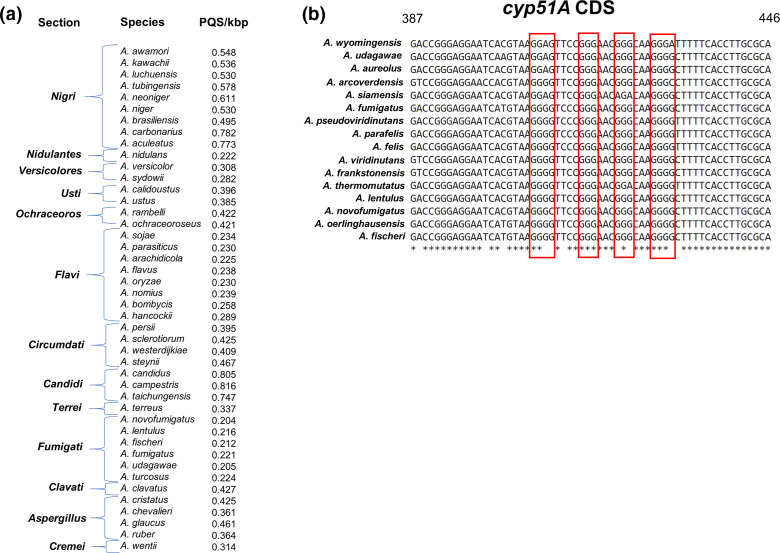
PQS in *Aspergillus* spp. are genetically conserved. (a) *Aspergillus* spp. were categorized into sections based upon a phylogenetic tree generated by Steenwyk *et al*. [46]. The frequency of PQS was shown to be closely associated with the genetic relatedness of these fungi. (b) A PQS was found in an exonic region of *cyp51A* from *A. fumigatus*. A blast search of this sequence identified conservation of this exact sequence or variations of this sequence within the section *Fumigati* (highlighted in red). Alignments were performed using the Clustal Omega web tool (EMBL-EBI). Sequence is reverse complemented.

The frequency of PQS/kbp appeared to be intrinsically linked to how closely related species were, with species within the same section displaying similar PQS frequencies (Fig. 5). Aspergilli were divided into 13 sections (range of PQS/kbp in brackets), the *Aspergillus* (0.364–0.461), the *Fumigati* (0.204–0.224), the *Candidi* (0.747–0.816), the *Circumdati* (0.395–0.467), the *Flavi* (0.225–0.289), the *Ochraceoros* (0.421–0.422), the *Usti* (0.385–0.396), the *Versicolores* (0.282–0.308) and the *Nigri* (0.495–0.782). The *Nigri* displayed the largest variation in PQS, however the lesser related species *A. carbonarius* and *A. aculeatus* skewed this range and if only the closest related species were noted, this range would be from 0.495 to 0.611 (Fig. 5a). As the *Nidulantes*, *Terrei* and *Clavati* only contained one member each, correlations in these sections could not be made. Notably, we found a sequence likely to form a quadruplex within *cyp51A* in *A. fumigatus,* and due to the important implications of *cyp51A* in azole resistance, we chose to see if this sequence was conserved within the section *Fumigati*. Interestingly, *A. fischeri*, *A. novofumigatus* and *A. lentulus* all retained the PQS observed in *A. fumigatus*. Moreover, this sequence was retained in cryptic *Aspergillus* spp. such as *A. felis* and *A. viridinutans*, whilst a variation on this sequence could be found in *A. arcoverdensis* (Fig. 5b). However, no sequence with significant similarity could be found in *A. turcosus* and the sequences in *A. udagawae, A. wyomingensis, A. siamensis* and *A. aureolus* would be unlikely to form the same quadruplex structure predicted for *A. fumigatus*.

### Loss of PQS may be associated with pathogenicity in *Aspergillus* spp

**Fig. 6.**
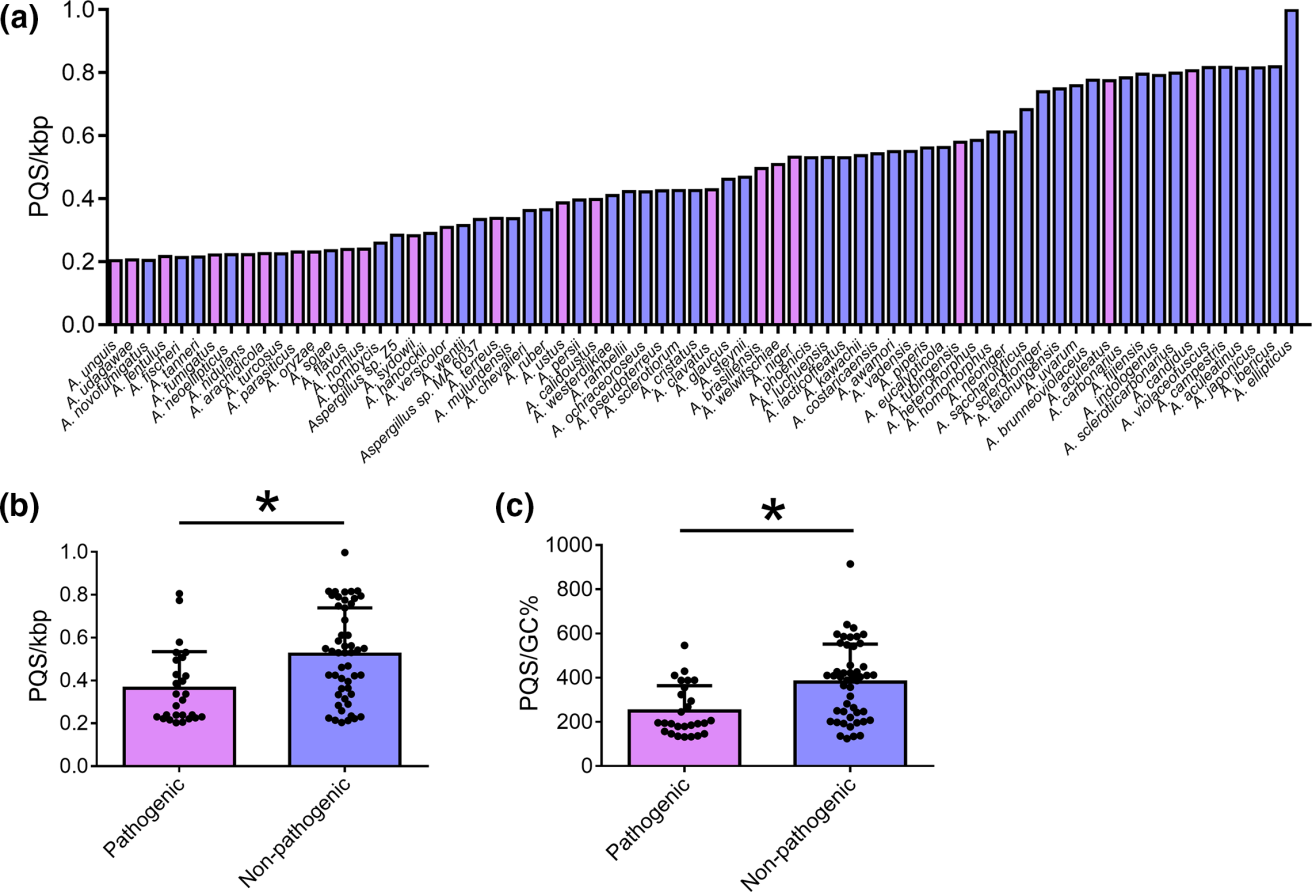
Pathogenic *Aspergillus* species have lower PQS frequencies compared to their non-pathogenic/infrequently pathogenic counterparts. The frequency of PQS/kbp and PQS/GC% were quantified and compared between species within *Aspergillus* spp. (a) The PQS/kbp in all species of *Aspergillus* in the study. The frequency of PQS/kbp (b) and PQS/GC% (c) in pathogenic and non-pathogenic/infrequently pathogenic species. Dots represent individual species within a genus. Pink bars represent pathogenic species, whilst blue bars represent non-pathogenic/infrequently pathogenic organisms. The error bars represent the sd. Significance was determined by Student’s *T* test. Asterisks indicate *P*<0.05.

The Ascomycota contains many of the most prevalent fungal pathogens of both plants and humans, including the genera *Aspergillus* spp. which contains the important human pathogen, *A. fumigatus*. Although, not all species within *Aspergillus* spp. are frequent causes of infection in humans and we found high variation in the PQS frequencies amongst species (Fig. 6a). Therefore, we compared the PQS frequency between species currently considered to be the most pathogenic and those which are non-pathogenic or infrequently pathogenic to humans, to explore whether there was a link between PQS-frequency and pathogenicity.

Indeed, comparing 72 species of *Aspergillus* (22 pathogenic, 50 non-pathogenic/infrequently pathogenic) pathogenic species had a significantly lower frequency of PQS/kbp (0.364 vs. 0.523) and PQS/GC% (249.9 vs. 380.5) within their genomes on average, compared to non-pathogenic species (Fig. 6b and c). This was an observation that was also noted for *Cryptococcus* spp. (containing the important basidiomycete pathogen *Cryptococcus neoformans*) but not for *Candida* spp. (containing the clinically relevant pathogen *Candida albicans*; Fig. S3). Furthermore, the most pathogenic species, *A. fumigatus,* had a lower than average frequency of PQS (0.221 PQS/kbp; Fig. 6a). Although PQS frequency within *A. fumigatus* was one of the lowest amongst aspergilli, this did not mean that PQS were not enriched within certain genomic regions or within certain genes. Therefore, we explored *A. fumigatus* in further detail, and endeavoured to identify the genetic location and potential functional roles of quadruplexes in this organism.

### PQS are enriched in repeat regions and mRNA, depleted in the tRNA, and are predominantly found in genes involved in metabolism in *A. fumigatus*

All previous wide-scale analyses were conducted using a stringent threshold of 1.5, as this highlighted sequences that were practically guaranteed to form quadruplexes. However, many sequences scoring below this threshold also have the potential to form these secondary structures. Therefore, the default G4Hunter threshold of 1.2 was used for the in-depth analyses of *A. fumigatus* to identify the entire repertoire of fungal PQS, as quadruplex formation for sequences above this threshold have been experimentally validated [53].

Genomic rearrangements have been suggested to contribute to the success of fungal pathogens, their ability to colonize and invade, and their adaptation to host niches. Repetitive genomic regions in *A. fumigatus* (e.g. coding tandem repeats) serve as hotspots for genome rearrangement, and mRNA splicing events in around 30 % of genes (~3000 genes) contribute to significant functional diversity, which likely contributes to the rapid adaptation to new environments [54, 55]. Quadruplexes have previously been found within several genomic regions in eukaryotes, including mRNA, introns, exons, promoters and telomeric regions. Evidence for their involvement in regulating processes such as mRNA splicing is ever increasing, and it is likely that quadruplexes could regulate these processes in fungi [56]. Therefore, we explored the genomic location of PQS within annotated genomic features in *A. fumigatus* to provide some insight into their potential functional roles.

To evaluate the position of PQS within *A. fumigatus*, the annotation information for the strain Af293 was obtained from the NCBI database and the presence of PQS within defined genomic features, or within 100 bp before or after these features were analysed. Both the total number of PQS per feature and the frequency of PQS/kbp of the described features were noted.

**Fig. 7.**
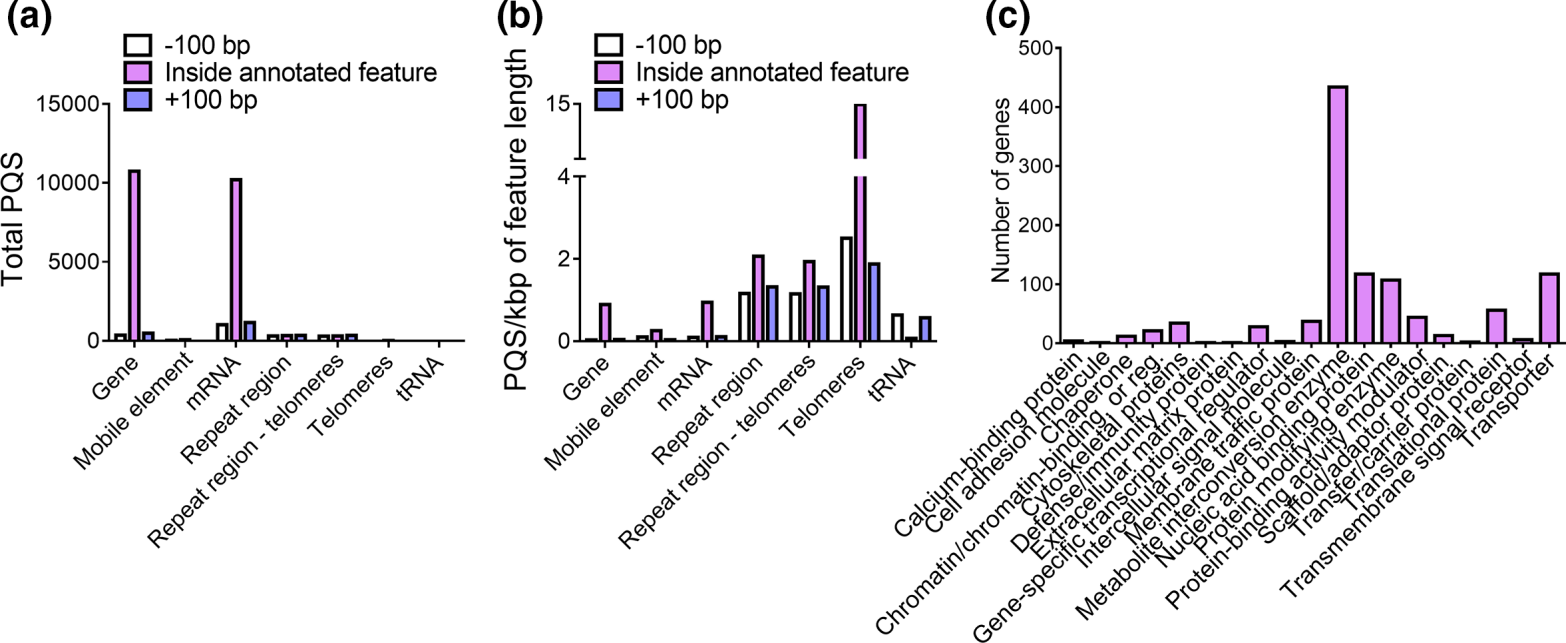
PQS in *A. fumigatus* are enriched in the repeat regions and mRNA. The location of PQS found 100 bp before, within, and 100 bp after annotated genomic features in *A. fumigatus* with a G4Hunter score ≥1.2 were identified. (a) The total number of PQS in known genomic features and (b) frequency of PQS comparative to the genomic length of the annotated features. (c) PQS-containing genes are primarily associated with metabolism. Associated protein classes were determined using PANTHER Protein Class v.15.0.

When only total PQS were considered, the largest number of PQS could be found within the genes and mRNA, with few PQS found in other genomic features (Fig. 7a). This was also the same in *C. albicans* and *C. neoformans* (Fig. S4). However, this was not the same when considering the frequency of PQS/kbp of the genomic features. In *A. fumigatus,* the greatest frequency of PQS could be found in the repeat regions and mRNAs, whilst the lowest frequency could be found within the tRNAs (Fig. 7b). It is well established that PQS are enriched in telomeres and the PQS frequency in *A. fumigatus* telomeres was high (14.95 PQS/kbp). However, PQS were also abundant in non-telomeric repeat regions (2.07 PQS/kbp in all repeat regions vs. 1.93 PQS/kbp in repeat regions without telomeres; Fig. 7b). Notably, tandem repeats found in *A. fumigatus* genes play important roles in host–pathogen interactions and contribute to a diverse range of functional proteins. Levdansky *et al.,* [55] highlighted 292 genes, which contained coding tandem repeats in the *A. fumigatus* genome. We explored these tandem repeat sequences and found that 130 (44.5 %) of these genes contained at least one PQS in their tandem repeat regions (Table S3). These observations were suggestive of the potential involvement of quadruplexes in the promotion of genetic diversity and adaptation in *A. fumigatus*.

In *A. fumigatus*, 35.1 % of genes contained at least one PQS likely to form quadruplex structures. In *C. neoformans*, this number was almost double, with 59.9 % of genes containing PQS. Conversely, PQS were only found in 5.6 % of genes in *C. albicans*. Despite the discrepancies in the number of genes where PQS can be found between the organisms, in all cases, PQS were primarily located in genes that encoded proteins involved in metabolism, nucleic acid binding, cell transport and protein modification (Fig. 7c). They were least likely to be found in genes encoding for calcium-binding proteins, extracellular matrix proteins, cell adhesion molecules and defence/immunity proteins.

### Quadruplexes may be involved in numerous important biological functions in *A. fumigatus* and genes upregulated in hyphae or during hypoxia are enriched in PQS

**Fig. 8.**
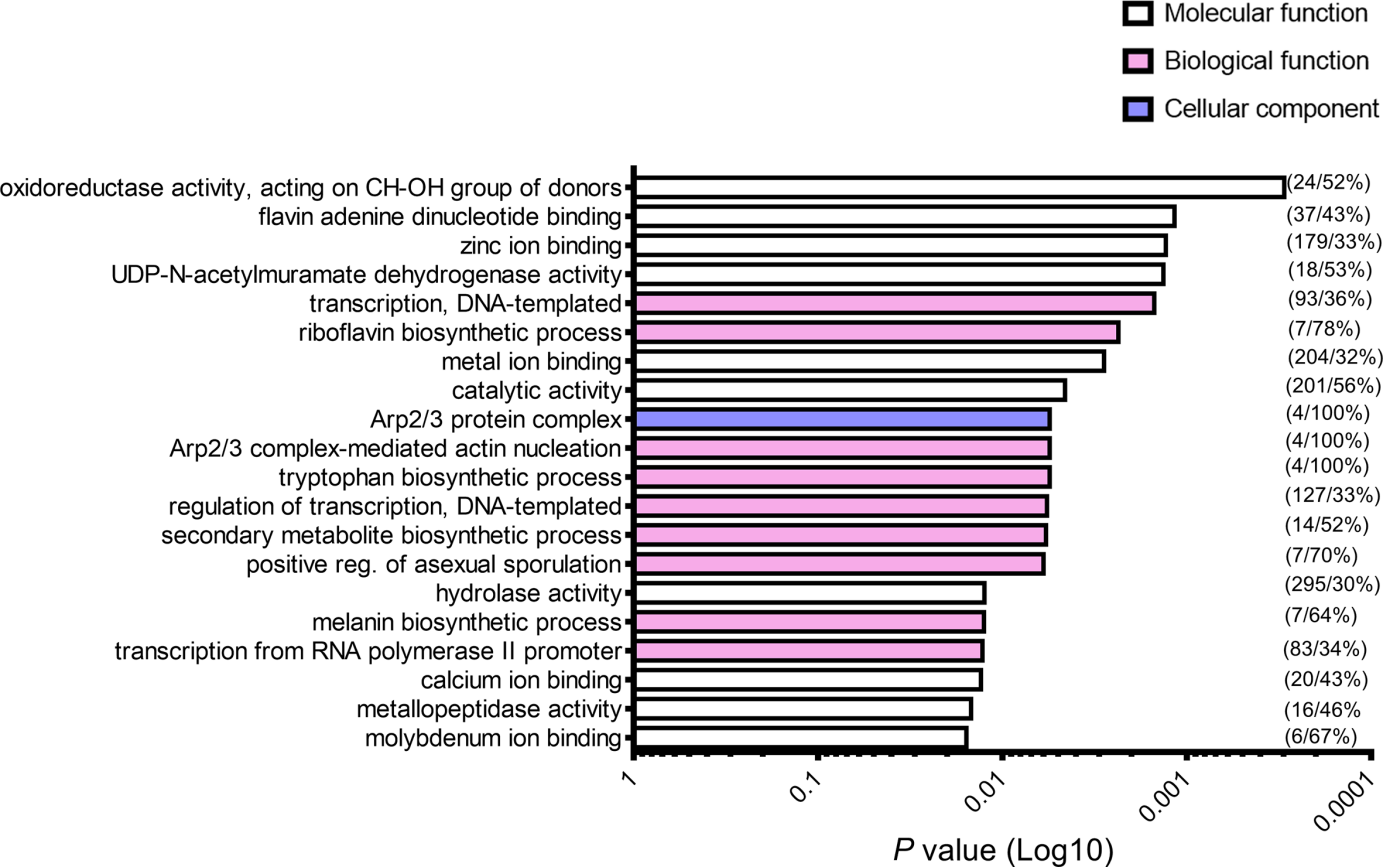
PQS-containing genes may be involved in numerous biological/molecular functions within pathogenic fungi. GO-term enrichment analysis of PQS-containing genes was performed using the FungiFun web tool V2.2.8. White bars indicate terms associated with molecular function, pink bars indicate terms associated with biological functions, and blue bars indicate terms associated with cellular components. The first number in brackets represents the number of genes and the second is the percentage of genes represented in the annotated category. Significance was determined via Fisher’s exact test. *P*<0.05 was considered significant.

As we knew the genomic location of the PQS, we could then identify the number and identity of the genes that contained these sequences. This further enabled us to identify their potential biological implications. GO-term analysis was conducted to identify the potential biological/molecular functions that PQS-containing genes might participate in. In *A. fumigatus*, we observed enrichment of genes involved in numerous functions such as oxidoreductase and hydrolase activity, zinc and molybdenum ion binding, transcription, and the biosynthesis of riboflavin, tryptophan, melanin and secondary metabolites (Fig. 8a). All the enriched terms can be found in Table S4.

**Fig. 9.**
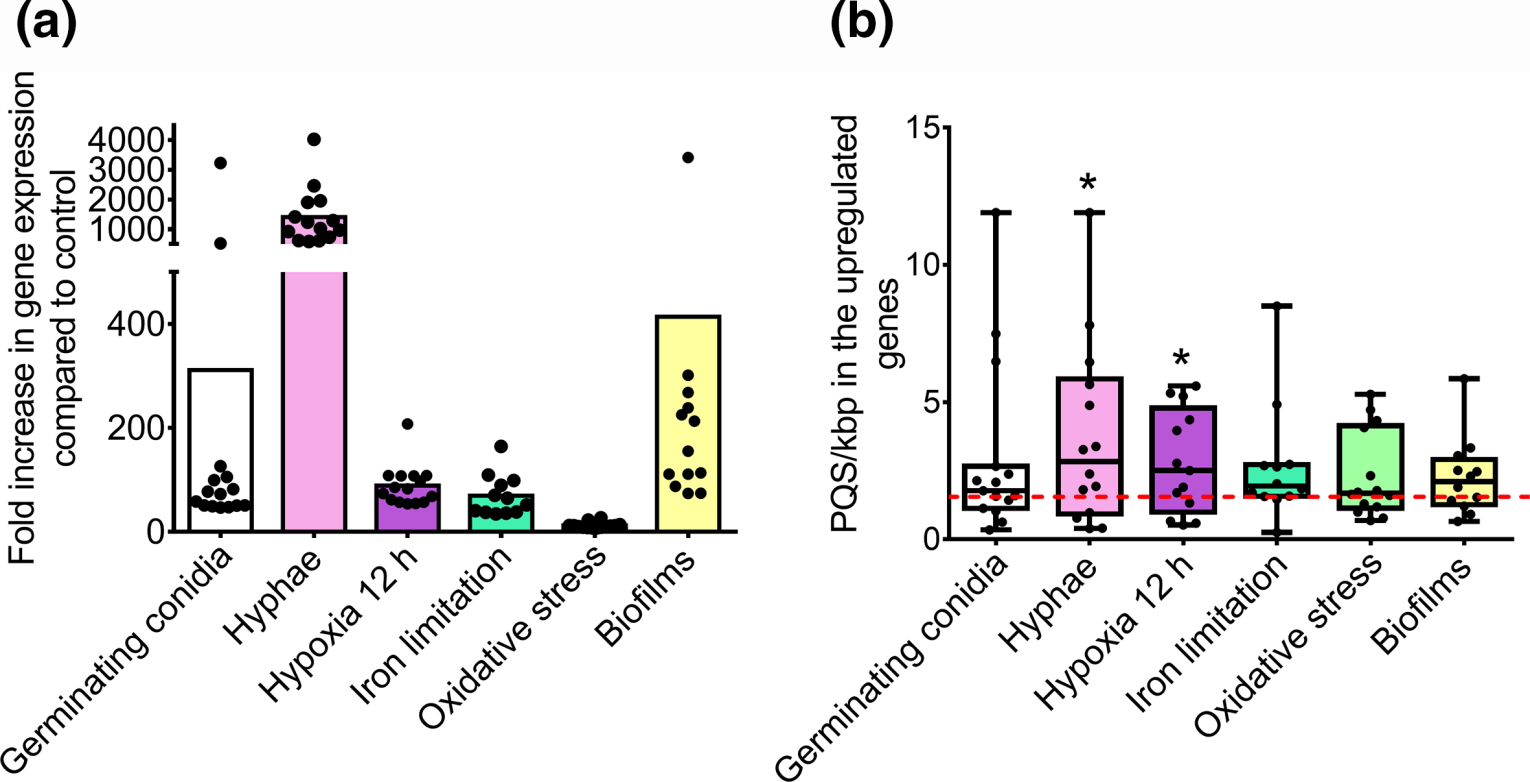
PQS can be found in genes that are highly upregulated during fungal germination, in response to environmental stresses, and in biofilms in *A. fumigatus*. Transcriptomes of *A. fumigatus* during germination, hypoxia, iron-limiting conditions, oxidative stress and in biofilms, were analysed and the 20 most upregulated genes were further investigated using QGRS Mapper and G4Hunter. (a) Genes that were highly upregulated (compared to dormant or untreated *A. fumigatus* conidia) contained PQS. Data points represent genes that contain PQS. (b) The average PQS frequency of genes upregulated in hyphae and during hypoxia were significantly higher when compared to the average PQS/kbp for the entire genome (1.55 PQS/kbp, red-dashed line), when analysed with the default G4Hunter settings. Data points represent the PQS frequency of an individual gene. Boxplot represents the median, maximum and minimum values. Whether the mean frequency of PQS within the upregulated genes of each condition was significantly higher compared to the mean of the entire genome was analysed via one-sample *T* test. * indicates a significant difference (*P*<0.05).

Although PQS could be found in genes associated with key fungal processes, we aimed to link the presence of PQS within these organisms with potential biological and pathophysiological functions. To investigate this, transcriptome datasets of *A. fumigatus* during germination [47], hypoxia [48], iron limitation and oxidative stress [49], or in biofilms [51] were analysed. In each instance, the top 20 upregulated genes (compared to dormant/unstressed *A. fumigatus* controls) were investigated for the presence and frequency of PQS. Notably, PQS could be found in 77.5 % of the genes investigated (Fig. 9a). This included genes that were upregulated in germinating conidia (16/20; 80.0 %), hyphae (14/20; 70.0 %), after 12 h of hypoxia (17/20; 85.0 %), following iron limitation (17/20; 85.0 %), undergoing oxidative stress (14/20; 70.0 %), and in biofilms (15/20; 75.0 %). These genes were investigated further to assess whether they were enriched with PQS and contained a higher frequency of PQS comparative to the average frequency found in the *A. fumigatus* genome. In all cases, the average PQS frequencies in the upregulated genes were higher than the average PQS observed throughout the entire genome (Fig. 9b, Table S5). However, only the average PQS frequencies in the hyphae and hypoxia groups were deemed significantly higher compared to the average genome frequency. Moreover, 71.4 % of genes upregulated in hyphae and 69.2 % upregulated in hypoxia contained PQS frequencies greater than the average in the genome. The genes containing the highest PQS frequencies in each condition were Afu8g01710 in germinating conidia and hyphae (11.90 PQS/kbp), Afu4g09580 in hypoxic fungi (5.59 PQS/kbp), Afu3g03650 during iron limitation (8.50 PQS/kbp), Afu5g10220 during oxidative stress (5.28 PQS/kbp) and Afu8g01980 in biofilms (5.90 PQS/kbp). Interestingly, each of these genes were upregulated in at least three out the six conditions investigated.

### PQS are in genes linked to virulence and drug resistance in *A. fumigatus*

G4s have been characterized in virulence genes from several pathogens and have arisen as a promising target for antimicrobial therapy and overcoming drug resistance [25]. Moreover, an iM in the promoter of the HIV-1 pro-viral genome has also been recently described [37]. Thus, whether PQS could be found in genes associated with virulence/drug resistance in *A. fumigatus* was explored.

Although the functions of many *A. fumigatus* genes are unknown, at least 56 genes have been found to be associated with the virulence and drug resistance of this organism (Table 1). PQS could be found in ~78 % of these genes (44/56 genes). This included genes that have been implicated in azole resistance (*cyp51A, cyp51B, abcC, hsp90, atrF, atrR, hmg1*) and echinocandin resistance (*fks1*). Genes implicated in virulence, which were enriched in PQS compared to the average frequency for the entire genome (PQS/kbp in brackets), included *mkk2* (4.78), *zafA* (4.04), *laeA* (2.36), *rasB* (4.97), *medA* (4.25), *glfA* (2.56), *pacC* (3.60), *sidG* (7.06), *hapX* (2.71), *calA* (7.44), *zrfC* (5.23), *aspf13* (8.65), *cpcA* (2.46), *gel2* (2.35) and *mscA* (2.63). Furthermore, numerous genes also contained high-scoring sequences likely to form quadruplexes, which were potentially composed of three or more G-tetrads; including, *zafA, pes1, laeA, pkac1, hasA, pacC, lysF, kre2, zrfC, hscA, mcsA, atrR, cyp51A, cyp51B, abcC, atrF* and *hmg1* (Table 1).

**Table 1.** PQS in genes associated with virulence and drug resistance in *A. fumigatus*

Identifier	Gene	Description	Sequence
Afu2g02690	*atrR*	Fungal specific transcription factor, putative	**GGGGG**A**GGG**TGC**GGG**ATGCTCTTC**GGG**
Afu4g06890	*cyp51A*	14-alpha sterol demethylase	**GGG**TCCC**GGG**AAC**GGG**CAA**GGGG**
Afu7g03740	*cyp51B*	14-alpha sterol demethylase	**GGGG**TA**GGGGGG**CCCA**GGG**
Afu1g14330	*abcC*	Azole transporter	**GGG**CCT**GGG**ACA**GGG**TCC**GGG**
Afu6g04360	*atrF*	Putative ABC transporter	**GGG**TATTTGTTT**GGGGGG**AT**GGG**
Afu5g04170	*hsp90*	Heat shock protein	**GG**A**GG**A**GG**A**GG**
Afu6g12400	*fks1*	Putative 1,3-beta-glucan synthase catalytic subunit, major subunit of glucan synthase	**GG**AA**GGGG**CTC**GGG**CAT**GGG**
Afu2g03700	*hmg1*	Hydroxymethylglutaryl-CoA (HMG-CoA) reductase	**GGG**C**GG**CA**GGG**ACA**GGGG**CAACTTC**GGG**
Afu1g05800	*mkk2*	Putative mitogen-activated protein kinase kinase (MAPKK)	**GG**TC**GG**T**GGG**T**GG**
Afu1g06900	*crzA*	C2H2-type zinc finger transcription factor involved in calcium ion homeostasis	**GG**AAT**GGG**A**GG**A**GGG**
Afu1g10080	*zafA*	Putative C2H2 zinc-responsive transcriptional activator	**GGG**CA**GGG**CAGAGCA**GGG**CA**GGGG**
Afu1g10380	*nrps1*	Non-ribosomal peptide synthetase (NRPS)	**GGG**AGCAA**GGGG**AAATCGCTGA**GGG**T**GGG**
Afu1g10880	*pmcA*	Putative P-type calcium ATPase	**GGG**C**GGG**TCAGAATCAGACTT**GGG**AA**GGG**
Afu1g13140	*gpaB*	G protein-coupled receptor alpha subunit	**GG**T**GG**A**GG**T**GG**
Afu1g14660	*laeA*	Involved in regulation of secondary metabolism	**GGG**ATGT**GGG**ACA**GGG**ATTT**GGG**
Afu1g17200	*sidC*	Fusarinine C non-ribosomal peptide synthetase (NRPS), putative	**GG**AGAA**GG**ATC**GGG**CAA**GG**
Afu2g07680	*sidA*	l-ornithine N5-oxygenase	**GG**CAAT**GG**CCA**GGG**AAGC**GG**
Afu2g07770	*rasB*	Ras family GTPase protein	**GGG**AC**GGGG**A**GGGG**C**GG**
Afu2g12200	*pkaC1*	cAMP-dependent protein kinase catalytic subunit	**GGG**CT**GGGGGG**TAA**GGGG**A**GGGGG**
Afu2g13260	*medA*	Putative regulator of adherence, host cell interactions and virulence	**GG**C**GG**A**GGG**AT**GG**
Afu2g17600	*pksP*	Conidial pigment polyketide synthase alb1	**GG**TT**GG**TC**GG**C**GGG**
Afu3g12890	*hasA*	C6 transcription factor hasA	**GGG**AA**GGG**AA**GGG**AA**GGG**
Afu3g12690	*glfA*	Putative UDP-galactopyranose mutase, enzyme in the first step of galactofuranose biosynthesis	**GG**TCTT**GGG**CTT**GG**TGTT**GG**
Afu3g11970	*pacC*	C2H2 finger domain transcription factor	**GGGGGGGGGG**AATGCTT**GGG**
Afu3g03650	*sidG*	Putative acetyltransferase with a predicted role in iron metabolism	**GG**AACT**GGG**TCT**GG**CCGA**GG**
Afu3g03420	*sidD*	Nonribosomal peptide synthetase 4	**GG**AA**GGGG**ACCC**GG**AC**GGGG**
Afu3g03400	*sidF*	Siderophore biosynthesis acetylase AceI, putative	**GG**ATC**GG**AT**GG**CGT**GG**
Afu3g02270	*cat1*	Mycelial catalase	**GG**ACGT**GG**AGT**GGG**AAT**GGG**
Afu5g03920	*hapX*	bZIP transcription factor HapX	**GG**AGAT**GG**AGAT**GG**AGAT**GG**
Afu5g08570	*pkaC2*	Class II protein kinase A (PKA)	**GGGGG**TC**GG**AA**GG**
Afu5g08890	*lysF*	Putative homoaconitase	**GGG**ACGC**GGGGGG**C**GG**CATC**GGG**AGC**GGG**
Afu3g09690	*calA*	Invasin calA	**GGG**CA**GGG**CAGT**GG**AGA**GGG**
Afu5g10760	*mnt1*	Putative alpha-1,2-mannosyltransferase with a predicted role in protein glycosylation	**GGG**AGA**GGG**CACA**GGGGGG**
Afu5g11230	*rasA*	Ras family GTPase protein	**GG**T**GG**T**GG**T**GG**
Afu4g09560	*zrfC*	Zinc transporter that functions in neutral or alkaline environments	**GGG**ATT**GG**T**GGGG**TCCA**GGGG**A**GGG**
Afu4g10460	*hcsA*	Homocitrate synthase, essential enzyme of the alpha-aminoadipate pathway of lysine biosynthesis	**GGG**T**GGGG**TTTATGA**GGG**C**GGG**
Afu2g12630	*aspf13*	Allergen Asp f 13	**GG**AT**GG**T**GGGGG**
Afu4g12470	*cpcA*	Transcriptional activator of the cross-pathway control system of amino acid biosynthesis	**GG**T**GG**C**GGGGG**
Afu6g09660	*gliP*	Nonribosomal peptide synthetase gliP	**GGG**AT**GG**CC**GGGG**AT**GG**
Afu6g11390	*gel2*	GPI-anchored 1,3-beta-glucanosyltransferase	**GG**TAC**GG**AGA**GG**ATGA**GG**
Afu8g02750	*cgrA*	Nucleolar rRNA processing protein	**GG**AAC**GGG**A**GGGG**A**GG**
Afu8g01670	*cat2*	Putative bifunctional catalase-peroxidase	**GG**TC**GGGG**TCTT**GG**TCCA**GG**
Afu6g04820	*pabA*	Para-aminobenzoic acid synthetase, an enzyme catalysing a late step in the biosynthesis of folate	**GG**T**GG**C**GGGGG**
Afu6g03590	*mcsA*	Methylcitrate synthase	**GGGG**TTC**GGG**TATTCGT**GGG**TCT**GGGG**

The highest scoring potential quadruplex-forming sequences for each of these genes were then re-analysed in an alternative PQS predictive algorithm called QGRS Mapper. The scores of sequences known to form quadruplexes were compared to scores of the PQS identified in *A. fumigatus* using this algorithm. This was conducted to provide further insight into whether these sequences were likely to form quadruplex structures. Known quadruplex-forming sequences were obtained from the G4IPDB database (*n*=94 sequences chosen at random) and were shown to have QGRS scores of around 21, 42 or 63 (depending on the number of G-tetrads – 2, 3 or 4, respectively). All the PQS-containing virulence genes contained at least one sequence scoring higher than 20, with many sequences scoring between 29 and 42 thus, very likely to form quadruplex structures, at least *in vitro* (Fig. S5).

### Altered PQS frequencies in orthologous genes in the close genetic relative of *A. fumigatus, A. fischeri*

*A. fischeri* is one of the closest genetic relatives of *A. fumigatus* and around ~90 % of the proteome is shared between the two species [57]. However, whilst *A. fumigatus* is regularly isolated from patients, only a few known cases of human infection with *A. fischeri* have ever been reported. Indeed, *A. fischeri* has been shown to be far less virulent compared to *A. fumigatus* in several murine models, but interestingly, *A. fischeri* appears to intrinsically display reduced susceptibility to azoles [57]. Furthermore, orthologues of all the *A. fumigatus* genes could be found in *A. fischeri.* Therefore, the PQS frequencies in orthologous genes in *A. fischeri* were quantified. Only quadruplex frequencies in the coding regions were compared between species, as other genetic regions of the *A. fischeri* orthologues are poorly understood.

**Fig. 10.**
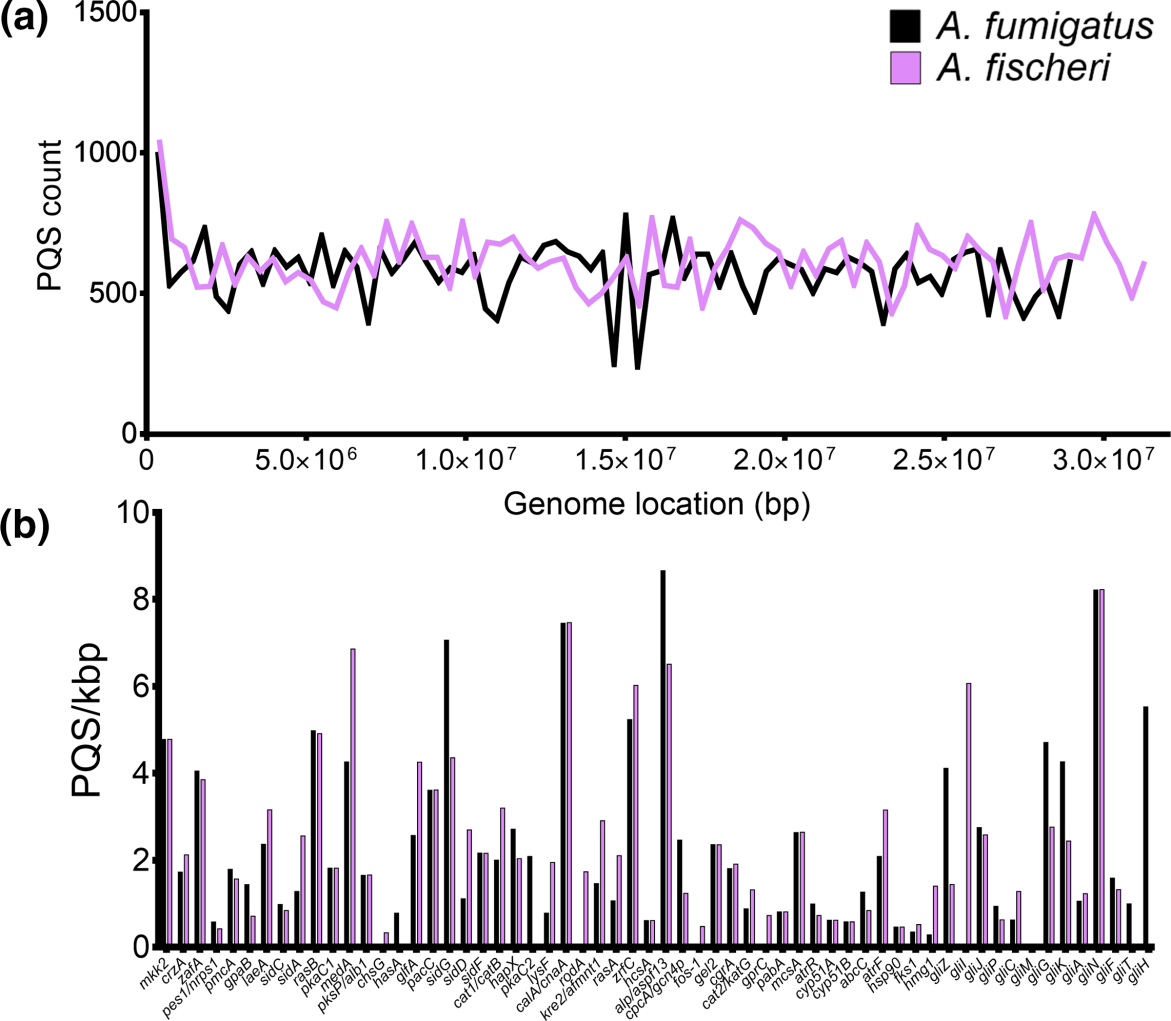
Differences between PQS frequency in genes involved in the pathogenicity of *A. fumigatus* and their orthologues in the close non-pathogenic relative, *A. fischeri* (a) The differences between the distribution of PQS in *A. fumigatus* (black) and *A. fischeri* (pink) genomes. (b) The PQS frequency in genes involved in virulence, drug resistance and gliotoxin biosynthesis in *A. fumigatus* (black), compared with the PQS frequency in the respective *A. fischeri* orthologues (pink). PQS frequencies were quantified using the default G4Hunter settings. The mean genome frequencies for *A. fumigatus* and *A. fischeri* were 1.55 PQS/kbp and 1.53 PQS/kbp, respectively.

Considering the genetic similarity between the two species and high sequence similarities in orthologues of the virulence genes under investigation (average similarities of 96.4 % and average sequence coverage of 99 %), the distribution of the PQS between the two appeared quite different (Fig. 10a). The PQS frequencies for *sidG, aspf13, cpcA, gliZ, gliG, gliK, gliT* and *gliH* were higher in *A. fumigatus* compared to *A. fischeri*. Conversely, the PQS frequencies for *laeA, sidA, medA, glfA, sidD, cat1, lysF, rodA, kre2, rasA, atrF, hmg1* and *gliI* were higher in *A. fischeri* compared to *A. fumigatus* (Fig. 10b, Table S6). Furthermore, the average PQS frequencies for the PQS-containing upregulated genes during hypoxia and in hyphae in *A. fumigatus* were higher in *A. fumigatus* when compared to *A. fischeri* orthologues (3.19 vs. 2.59 PQS/kbp and 3.65 vs. 3.00 PQS/kbp, for genes upregulated during hypoxia and in hyphae, respectively; Fig. S6). Moreover, *A. fischeri* orthologues of *hmg1, pkaC1, hasA, glfA, kre2* and *hscA* also do not share the high-scoring PQS that were found in *A. fumigatus*. The implication of these differences to the functions of these organisms, and whether the altered PQS frequencies are associated with virulence, are currently unknown. However, future studies investigating these differences might unveil how these species differ in their pathogenicity.

## Discussion

In this study, we computationally predicted PQS within the genomes of fungi. We made several important observations. This was the first study to investigate the frequency of PQS amongst numerous genetically distinct fungal species. Moreover, we highlighted the genomic location of PQS in *A. fumigatus*, their potential involvement in key biological processes, and discussed their presence in genes associated with virulence and drug resistance.

The frequency of PQS throughout genomes is highly variable; for example, human genomes were shown to contain around 0.228 PQS/kbp, whereas the genomes of *Escherichia coli* contain around 0.028 PQS/kbp [15]. In this study we also found significant differences in the number and frequency of PQS throughout fungal genomes. For example, fungi from the Saccharomycotina (primarily composed of yeasts) contained a low frequency of PQS. Conversely, filamentous fungi from the Pezizomycotina (containing many important pathogens of both plants and humans) had much higher PQS frequencies and >15 fold more PQS than fungi from the Saccharomycotina on average. It has previously been shown that G4s contribute to genetic instability in yeasts (*Saccharomyces* spp*.*) and as suggested for bacteria, it is possible that G4s may also have been deselected through evolution in ascomycete yeasts [58, 59]. However, things are more complicated concerning basidiomycetous yeasts such as *Cryptococcus* spp. and *Malassezia* spp. Cryptococcal species such as *C. wingfieldii, C. amylolentus* and *C. floricola* possessed some of the highest PQS frequencies in the study. Alternatively, all the *Malassezia* species investigated had much lower PQS frequencies than expected, which was surprising given their genomes were GC rich compared to many other fungi (>55 % GC content). We also identified many PQS within genes, and mRNA of *A. fumigatus,* but a higher frequency of PQS within the repeat regions and mRNA. It has previously been observed that *S. cerevisiae* promoters and open reading frames are enriched with sequences capable of potentially forming intramolecular G4s [60]. A recent study by Čutová *et al.,* also demonstrated that inverted repeats were enriched in the centromeres and rDNA/rRNA regions, and G4s were enriched in the telomeres and tRNAs of *S. cerevisiae* [61].

Notably, the genomes of pathogenic aspergilli and cryptococci contained significantly lower PQS frequencies relative to their non-pathogenic/infrequently pathogenic counterparts. Conversely, there was no correlation between PQS frequency and pathogenicity between *Candida* species or within the Saccharomycotina. Interestingly, loss of PQS has recently also been observed in pathogenic *Coronaviridae* [62]. The exact reasons underlying the loss of PQS are not currently known. Fungi may have evolved to retain the most essential quadruplex-forming sequences or those that provided them with a pathogenic advantage. It has been reported that host nucleolin (an RNA-binding protein) can bind and stabilize quadruplexes in the long terminal repeat (LTR) promoter of HIV-1, which can silence viral transcription [63]. Therefore, in this situation, loss of quadruplexes would be beneficial for immune evasion. Contrarily, it has been suggested that iMs within HIV-1 are triggered after they promote the acidification of intracellular compartments, modulating viral processes [37]. In this instance, the formation of quadruplexes could be beneficial and promote pathogenicity. In the case of *Aspergillus* and *Cryptococcus* species, this loss of PQS might also be linked with evasion of the immune response. Furthermore, the low PQS frequency observed within *Candida* and *Malassezia* species might be associated with their ability to form commensal relationships and avoid stimulating a host response. The importance of quadruplex stabilization may therefore be situational and could be detrimental or beneficial to the fungus depending on the environment. It is known that fungal pathogens such as *A. fumigatus* and *C. neoformans* can survive the acidic conditions within intracellular organelles to propagate infection [64, 65]. Therefore, it could be interesting to investigate the dynamics/flux of quadruplex formation under conditions that fungi would experience within the host and whether these structures can contribute to fungal survival and propagation.

Although the overall genome PQS frequency in *A. fumigatus* was one of the lowest amongst the aspergilli, the average PQS frequencies in genes involved in virulence and gliotoxin synthesis, or those upregulated in response to conditions such as hypoxia, iron limitation and oxidative stress, appeared to be much higher than the average genome frequency. GO-term enrichment analysis implicated potential roles for PQS-containing genes in functions associated with virulence in pathogenic fungi such as zinc-ion binding [66], riboflavin and tryptophan biosynthesis [67], melanin [68] and secondary metabolite synthesis [69], metallopeptidase activity [70], and the response to environmental stimuli [71]. Moreover, PQS were predominantly found in genes associated with fungal metabolism.

It is widely acknowledged that metabolism in fungi is central to its virulence, pathogenicity and survival [72]. The ability of a fungus to rapidly adapt to the host microenvironment is achieved through processes such as metabolic remodelling, stress resistance and the utilization of amino acids [67]. This in turn has a significant effect on the triggering of important virulence traits, like the production of hyphae, biofilm growth, capsule formation in *C. neoformans* and melanization [67]. Moreover, metabolic pathways influence fungal vulnerability to innate immune defences and can regulate susceptibility to antifungal drugs [73]. Not only primary metabolism, secondary metabolism results in the release of various secondary metabolites and toxins, such as gliotoxin, aflatoxin and candidalysin [74, 75]. Furthermore, many genes containing PQS within *S. cerevisiae,* humans and bacteria were also primarily associated with metabolism [76, 77]. These data support the observations made in *A. fumigatus* and implicates an important role for quadruplexes in metabolism throughout different organisms. Thus, the enrichment of PQS within these genes is suggestive of the potential importance of quadruplexes in the regulation of key biological functions and stress responses, and it would be interesting to explore the effects quadruplex-targeting ligands could have on these processes.

There are now an ever-increasing number of G4s identified within genes linked to microbial pathogenicity. G4-forming motifs located in the *hsdS, recD* and *pmrA* genes of *S. pneumoniae,* and *var* genes of *P. falciparum* can modulate host–pathogen interactions [78, 79]. Moreover, targeting G4s in *espK, espB* and *cyp51* from *Mycobacterium tuberculosis* with the G4/iM-binding ligand TMPyP4 could inhibit transcription of these essential virulence genes [80]. Binding of several G4-targeting ligands to G4s in viruses have been shown to limit the virulence of the Herpes simplex virus-1, HIV-1, Ebola and Hepatitis C [81]. Interestingly, dinuclear ruthenium (II) complexes (well-characterized G4 and iM DNA binding agents) are active against methicillin-resistant *S. aureus* and vancomycin-resistant *Enterococcus* spp. [35, 36], and another G4/iM-ligand, berberine, has also demonstrated activity towards fluconazole-resistant *C. albicans* and *C. neoformans* [82].

Notably, sequences highly likely to form quadruplexes (G_3_+L_1-12_) were also found in important genes implicated in azole resistance (*atrR, cyp51A, cyp51B, abcC* and *hmg1*) and virulence (*zafA, pes1, laeA, pkac1, hasA, pacC, lysF, kre2, zrfC, hscA* and *mcsA*). Many of which were not found to be shared with the close non-pathogenic relative, *A. fischeri*. Mutations in *cyp51A* are a major contributing factor to azole resistance, but there is growing evidence that mutations in *cyp51B* and the involvement other members of the ergosterol synthesis pathway contribute to this resistance phenotype [83, 84]. Moreover, genes such as *laeA* (encoding a master regulator of secondary metabolite biosynthesis) and *pacC* (encoding a pH-responsive regulator), regulate key virulent processes, such as gliotoxin production and host-cell adaption, respectively [85–87]. Thus, if the quadruplex structures in these genes form *in vivo* and if they are implicated in regulating their transcription, they could pose an interesting target to modulate fungal pathogenic processes, as observed for other pathogenic microorganisms, such as viruses.

## Conclusions

Here, we provide a representative overview of potential quadruplex-forming sequences across the fungal kingdom and discuss their potential implications in fungal virulence and drug resistance. If these structures form *in vivo* and are involved in regulating transcription and biological processes in fungi, then they could have the potential to represent novel antifungal targets. Understanding whether quadruplexes could form under pathophysiological conditions, whether fungi themselves could release quadruplex-binding ligands to modulate host responses, or identifying whether there are fungal specific quadruplex-forming sequences could also be important factors to consider. Thus, targeting quadruplexes in fungi could represent a novel antifungal target, although experimental validation is necessary.

## Supplementary Data

Supplementary material 1

Supplementary material 2
