# Supplementary material for: Analysis of putative quadruplex-forming sequences in fungal genomes: novel antifungal targets?
Source: Microb Genom. 2021 May 6;7(5):000570. doi: 10.1099/mgen.0.000570 (PMC8209732; doi:10.1099/mgen.0.000570)
Supplement: Supplementary material 2 [file mgen-7-0570-s002.pdf]

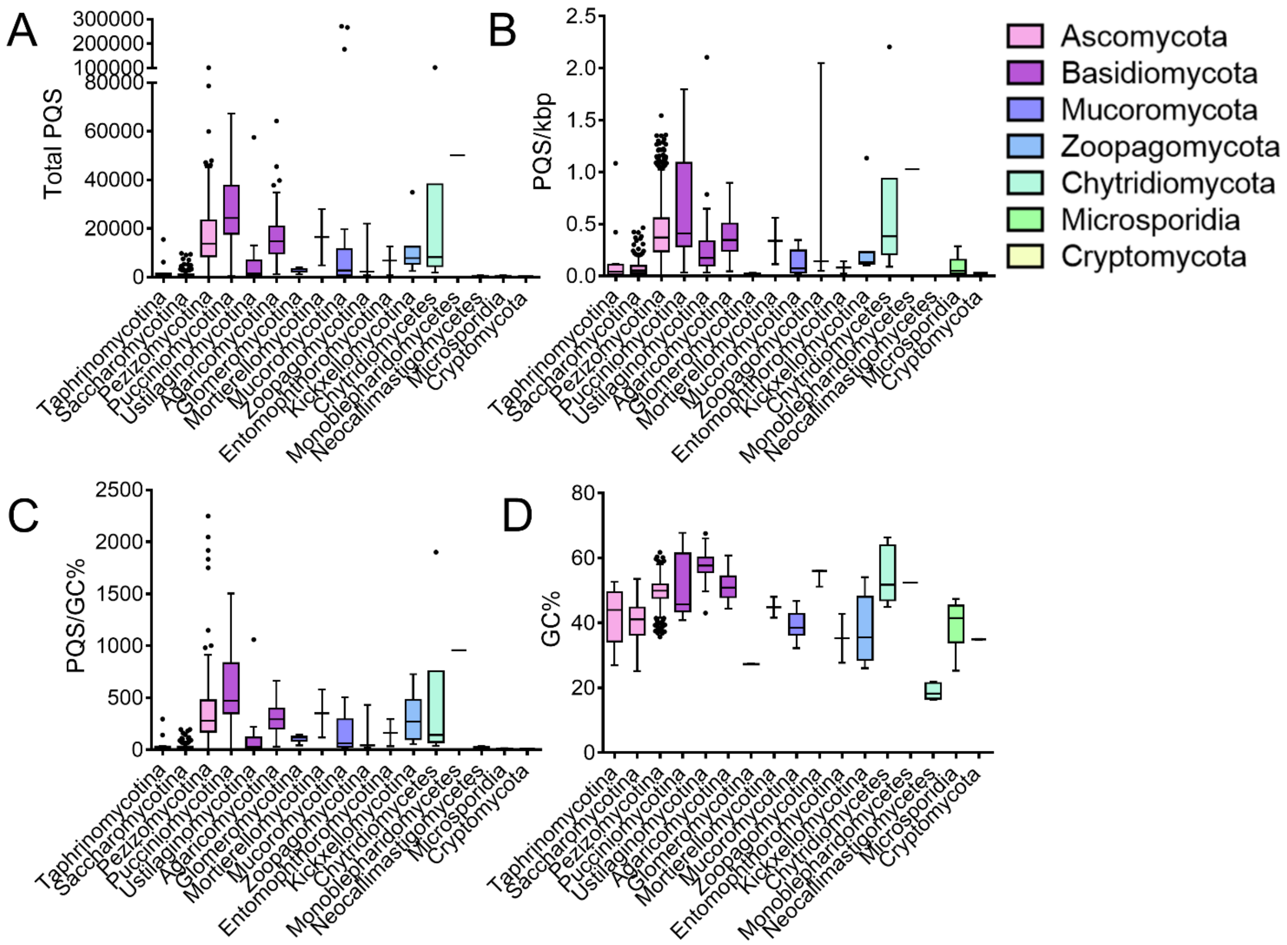

Supplementary Figure 1. Heterogeneity of PQS across fungal sub-divisions. The total number and frequency of PQS within fungal sub-divisions were quantified using G4Hunter with a threshold of 1.5 and window size of 30. The average number of PQS **(A)**, PQS/kbp **(B)**, PQS/GC% **(C)** and GC content **(D)** in fungi from the Taphrinomycotina (n=14), Saccharomycotina (n=332), Pezizomycotina (n=761), Pucciniomycotina (n=24), Ustilagomycotina (n=30), Agaricomycotina (n=132), Glomeromycotina (n=9), Mortierellomycotina (n=2), Mucoromycotina (n=20), Zoopagomycotina (n=3), Entomophthoromycotina (n=2), Kickxellomycotina (n=7), Chytridiomycetes (n=6), Monoblepharidomycetes (n=1), Neocallimastigomycetes (n=5), Microsporidia (n=9), and Cryptomycota (n=2). A to D contain boxplots with Tukey whiskers. The outliers are indicated by dots and the line within the boxplot is representative of the median value.

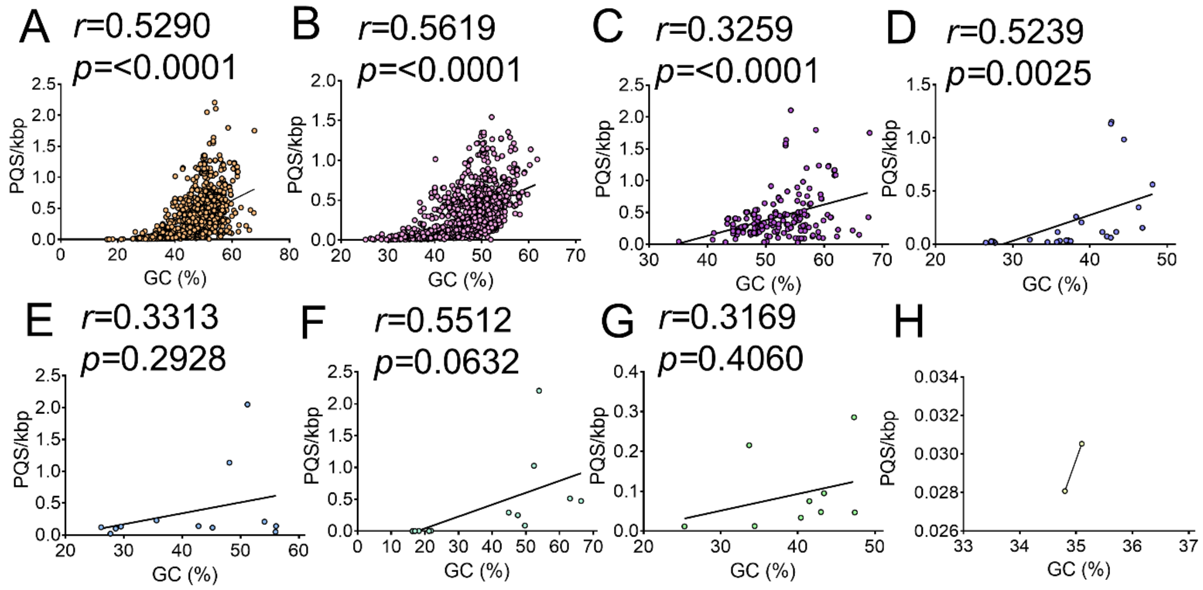

Supplementary Figure 2. Higher genome GC-content is positively correlated with the frequency of PQS. The frequency of PQS relative to the GC content of fungi was plotted for (A) all fungal genomes in the study, (B) the Ascomycota, (C) the Basidiomycota, (D) the Mucoromycota, (E) the Zoopagomycota, (F) the Chytridiomycota, (G) the Microsporidia, and (H) the Cryptomycota. The Pearson correlation coefficient was used to determine the association between PQS and GC content. $P<0.05$ was considered statistically significant.

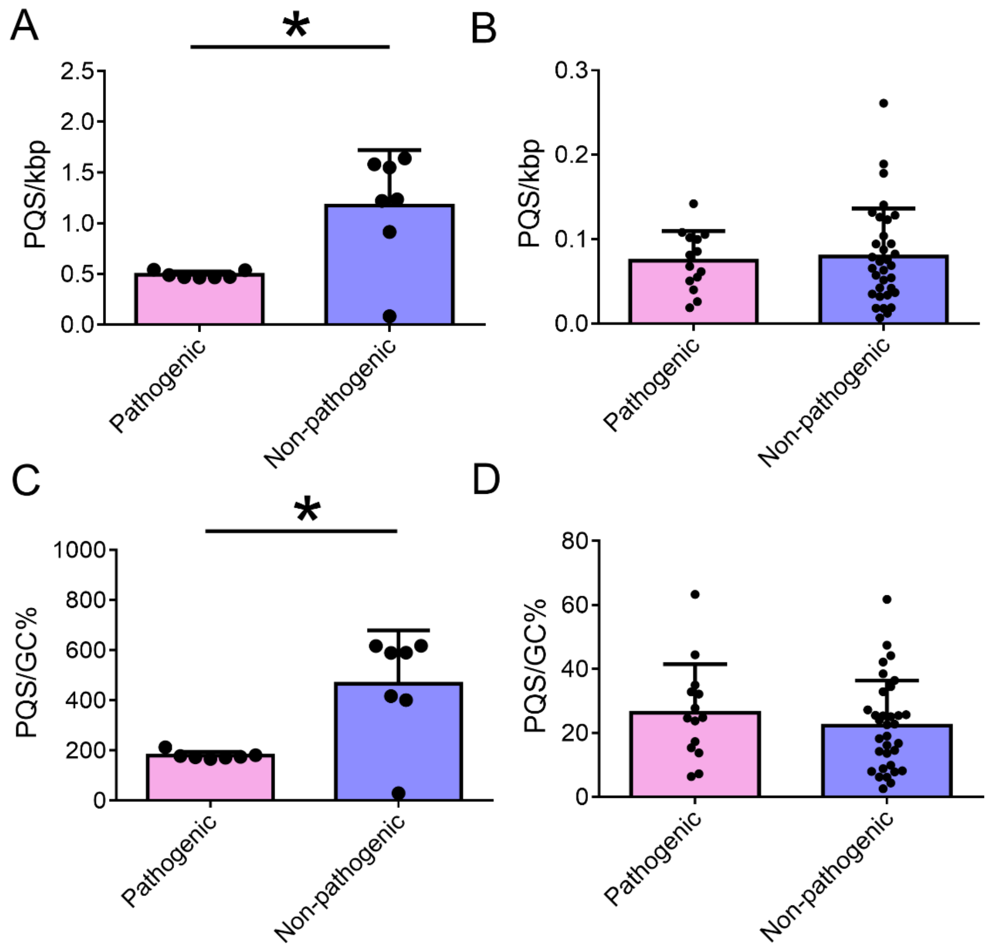

Supplementary Figure 3. Pathogenic *Cryptococcus* species have lower PQS frequencies compared to non-pathogenic/infrequently pathogenic species. The frequency of PQS/kbp and PQS/GC% were quantified and compared between pathogenic and non-pathogenic/infrequently pathogenic species within *Cryptococcus* spp., and *Candida* spp. The PQS/kbp in pathogenic and non-pathogenic/infrequently pathogenic species of **(A)** *Cryptococcus* spp., and **(B)** *Candida* spp. The PQS/GC% in pathogenic and non-pathogenic/infrequently pathogenic species of **(C)** *Cryptococcus* spp., and **(D)** *Candida* spp. Dots represent individual species within a genus. The error bars represent the SD. Asterisks indicate $p<0.05$.

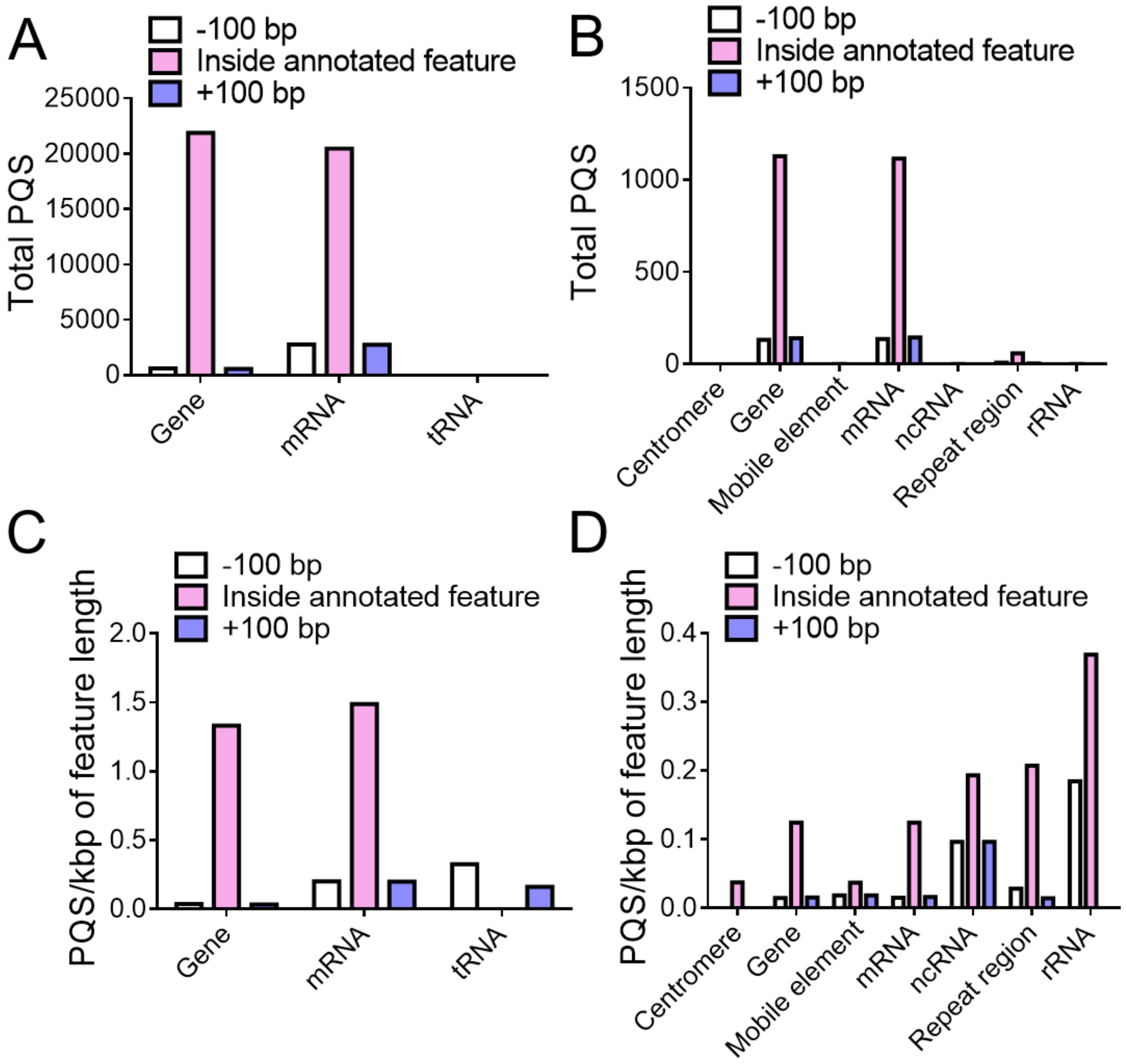

Supplementary Figure 4. PQS in *C. neoformans* JEC21, and *C. albicans* SC5314 can be found located throughout annotated genomic features. The location of PQS found 100 bp before, within, and 100 bp after annotated genomic features with a G4Hunter score >1.2. The total number of PQS in known genomic features in **(A)** *C. neoformans*, and **(B)** *C. albicans*. The frequency of PQS comparative to the genomic length of the annotated features in **(C)** *C. neoformans*, and **(D)** *C. albicans*.

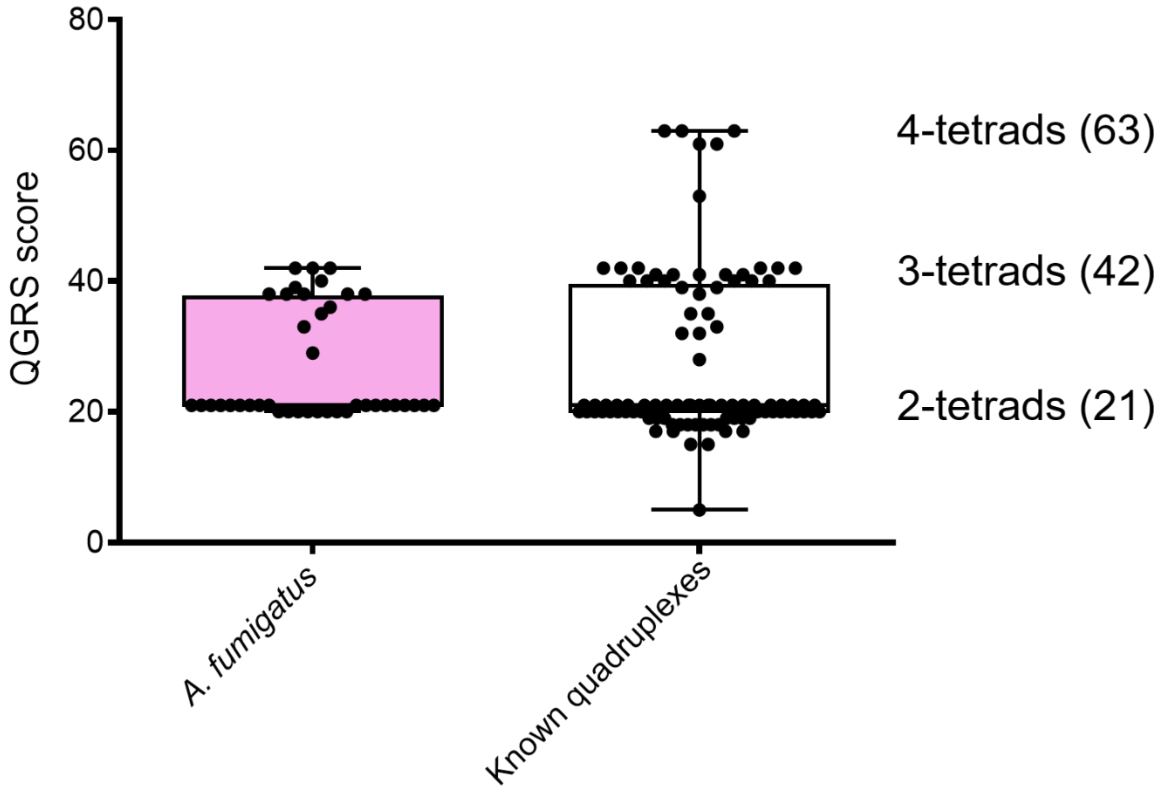

Supplementary Figure 5. The scores of PQS found in fungi compared to known quadruplex forming sequences. PQS in the identified genes were scored in QGRS Mapper and compared against the scores of known quadruplexes (n=94) to predict the propensity of PQS sequences to form quadruplex structures. Sequences with scores of 21, 42, and 63 in QGRS Mapper could form G4s containing 2, 3, and 4-tetrads, respectively. Sequences containing $G_{2+}L_{1-12}$ generally produced scores of 20 or 21, while those containing $G_{3+}L_{1-12}$ produced scores between 29-42.

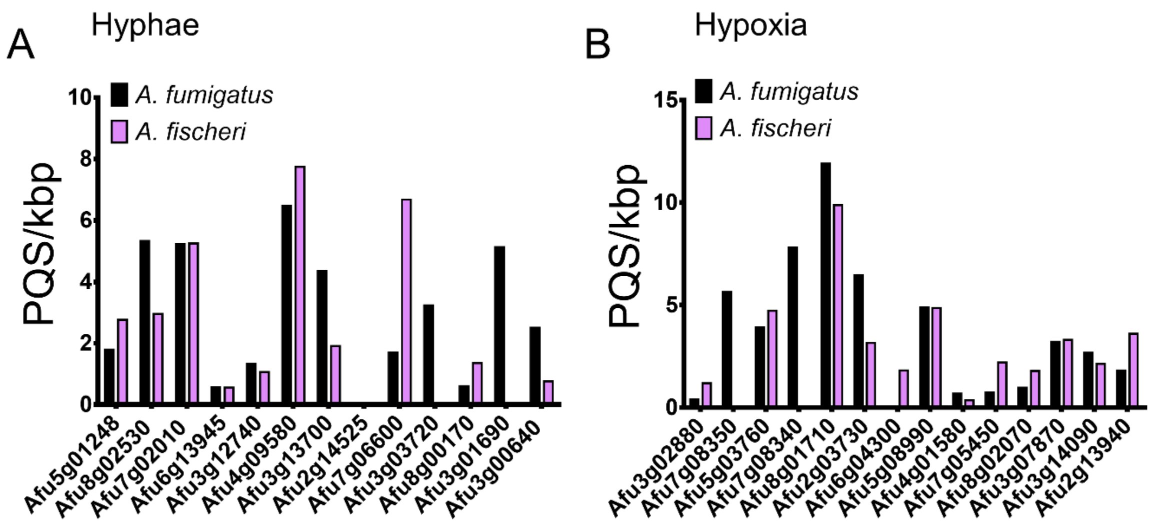

Supplementary Figure 6. The differences between PQS frequencies in the CDS of the genes upregulated in hyphae and during hypoxia in *A. fumigatus* and the orthologues of these genes in *A. fischeri*. The frequency of PQS in the CDS of genes upregulated in *A. fumigatus* in hyphae and during hypoxia were compared to the frequency of PQS in their orthologues in *A. fischeri*. PQS frequencies were determined using the default G4Hunter settings.
